# Large‐scale and long‐term wildlife research and monitoring using camera traps: a continental synthesis

**DOI:** 10.1111/brv.13152

**Published:** 2025-01-17

**Authors:** Tom Bruce, Zachary Amir, Benjamin L. Allen, Brendan F. Alting, Matt Amos, John Augusteyn, Guy‐Anthony Ballard, Linda M. Behrendorff, Kristian Bell, Andrew J. Bengsen, Ami Bennett, Joe S. Benshemesh, Joss Bentley, Caroline J. Blackmore, Remo Boscarino‐Gaetano, Lachlan A. Bourke, Rob Brewster, Barry W. Brook, Colin Broughton, Jessie C. Buettel, Andrew Carter, Antje Chiu‐Werner, Andrew W. Claridge, Sarah Comer, Sebastien Comte, Rod M. Connolly, Mitchell A. Cowan, Sophie L. Cross, Calum X. Cunningham, Anastasia H. Dalziell, Hugh F. Davies, Jenny Davis, Stuart J. Dawson, Julian Di Stefano, Christopher R. Dickman, Martin L. Dillon, Tim S. Doherty, Michael M. Driessen, Don A. Driscoll, Shannon J. Dundas, Anne C. Eichholtzer, Todd F. Elliott, Peter Elsworth, Bronwyn A. Fancourt, Loren L. Fardell, James Faris, Adam Fawcett, Diana O. Fisher, Peter J. S. Fleming, David M. Forsyth, Alejandro D. Garza‐Garcia, William L. Geary, Graeme Gillespie, Patrick J. Giumelli, Ana Gracanin, Hedley S. Grantham, Aaron C. Greenville, Stephen R. Griffiths, Heidi Groffen, David G. Hamilton, Lana Harriott, Matthew W. Hayward, Geoffrey Heard, Jaime Heiniger, Kristofer M. Helgen, Tim J. Henderson, Lorna Hernandez‐Santin, Cesar Herrera, Ben T. Hirsch, Rosemary Hohnen, Tracey A. Hollings, Conrad J. Hoskin, Bronwyn A. Hradsky, Jacinta E. Humphrey, Paul R. Jennings, Menna E. Jones, Neil R. Jordan, Catherine L. Kelly, Malcolm S. Kennedy, Monica L. Knipler, Tracey L. Kreplins, Kiara L. L'Herpiniere, William F. Laurance, Tyrone H. Lavery, Mark Le Pla, Lily Leahy, Ashley Leedman, Sarah Legge, Ana V. Leitão, Mike Letnic, Michael J. Liddell, Zoë E. Lieb, Grant D. Linley, Allan T. Lisle, Cheryl A. Lohr, Natalya Maitz, Kieran D. Marshall, Rachel T. Mason, Daniela F. Matheus‐Holland, Leo B. McComb, Peter J. McDonald, Hugh McGregor, Donald T. McKnight, Paul D. Meek, Vishnu Menon, Damian R. Michael, Charlotte H. Mills, Vivianna Miritis, Harry A. Moore, Helen R. Morgan, Brett P. Murphy, Andrew J. Murray, Daniel J. D. Natusch, Heather Neilly, Paul Nevill, Peggy Newman, Thomas M. Newsome, Dale G. Nimmo, Eric J. Nordberg, Terence W. O'Dwyer, Sally O'Neill, Julie M. Old, Katherine Oxenham, Matthew D. Pauza, Ange J. L. Pestell, Benjamin J. Pitcher, Christopher A. Pocknee, Hugh P. Possingham, Keren G. Raiter, Jacquie S. Rand, Matthew W. Rees, Anthony R. Rendall, Juanita Renwick, April Reside, Miranda Rew‐Duffy, Euan G. Ritchie, Chris P. Roach, Alan Robley, Stefanie M. Rog, Tracy M. Rout, Thomas A. Schlacher, Cyril R. Scomparin, Holly Sitters, Deane A. Smith, Ruchira Somaweera, Emma E. Spencer, Rebecca E. Spindler, Alyson M. Stobo‐Wilson, Danielle Stokeld, Louise M. Streeting, Duncan R. Sutherland, Patrick L. Taggart, Daniella Teixeira, Graham G. Thompson, Scott A. Thompson, Mary O. Thorpe, Stephanie J. Todd, Alison L. Towerton, Karl Vernes, Grace Waller, Glenda M. Wardle, Darcy J. Watchorn, Alexander W. T. Watson, Justin A. Welbergen, Michael A. Weston, Baptiste J. Wijas, Stephen E. Williams, Luke P. Woodford, Eamonn I. F. Wooster, Elizabeth Znidersic, Matthew S. Luskin

**Affiliations:** ^1^ Wildlife Observatory of Australia (WildObs), Queensland Cyber Infrastructure Foundation (QCIF) Brisbane Queensland 4072 Australia; ^2^ School of the Environment University of Queensland Brisbane Queensland 4072 Australia; ^3^ Terrestrial Ecosystem Research Network University of Queensland Brisbane Queensland 4072 Australia; ^4^ Centre for Sustainable Agricultural Systems, Institute for Life Sciences and the Environment University of Southern Queensland Toowoomba Queensland 4350 Australia; ^5^ Centre for African Conservation Ecology Nelson Mandela University Gqeberha 6034 South Africa; ^6^ Centre for Ecosystem Science, School of Biological, Earth and Environmental Sciences University of New South Wales Sydney New South Wales 2052 Australia; ^7^ Biosecurity Queensland, Department of Agriculture and Fisheries 203 Tor St Toowoomba Queensland 4350 Australia; ^8^ Queensland Parks and Wildlife Service, Department of Environment Science and Innovation PO Box 3130 Red Hill Queensland 4701 Australia; ^9^ School of Environmental and Rural Science University of New England Armidale New South Wales 2351 Australia; ^10^ Queensland Parks and Wildlife Service, Department of Environment Science and Innovation PO Box 101 Maryborough Queensland 4650 Australia; ^11^ University of Queensland Brisbane Queensland 4072 Australia; ^12^ Landscape South Australia, SA Arid Lands Landscape Board 1 Jervois St Port Augusta South Australia 5700 Australia; ^13^ School of Life and Environmental Sciences, Deakin University Burwood Campus, 221 Burwood Highway Burwood Victoria 3125 Australia; ^14^ Vertebrate Pest Research Unit, NSW Department of Primary Industries and Regional Development Orange Agricultural Institute 1447 Forest Road, Orange New South Wales 2800 Australia; ^15^ School of Agriculture, Food and Ecosystem Sciences The University of Melbourne Parkville Victoria 3010 Australia; ^16^ National Malleefowl Recovery Group 52 Naroon Rd Alphington Victora 3078 Australia; ^17^ Biodiversity, Conservation and Science, NSW Department of Climate Change, Energy, the Environment and Water 4 Parramatta Square, 12 Darcy Street Parramatta New South Wales 2150 Australia; ^18^ Rewilding Australia Program WWF‐Australia PO Box 528 Sydney New South Wales 2001 Australia; ^19^ School of Natural Sciences University of Tasmania, Sandy Bay Tasmania 7001 Australia; ^20^ Bush Heritage Australia , Level 10, 637 Flinders Street Docklands Victoria 3008 Australia; ^21^ Gulbali Institute Charles Sturt University Thurgoona New South Wales 2640 Australia; ^22^ Vertebrate Pest Research Unit, NSW Department of Primary Industries and Regional Development 11 Farrer Place Queanbeyan New South Wales 2620 Australia; ^23^ School of Science University of New South Wales, Northcott Drive Canberra Australian Capital Territory 2601 Australia; ^24^ South Coast Region, Parks and Wildlife Service, Department of Biodiversity Conservation and Attractions South Coast Region, 120 Albany Hwy Albany Western Australia 6330 Australia; ^25^ University of Western Australia, Centre of Excellence in Natural Resource Management Albany Western Australia 6330 Australia; ^26^ Evolution and Ecology Research Centre, School of Biological, Earth and Environmental Sciences University of New South Wales Sydney New South Wales 2052 Australia; ^27^ Global Wetlands Program, Coastal and Marine Research Centre, Australian Rivers Institute, School of Environment and Science Griffith University Gold Coast Queensland 4222 Australia; ^28^ School of Agriculture and Environmental Sciences University of Western Australia Crawley Western Australia 6009 Australia; ^29^ ARC Centre for Mine Site Restoration, School of Molecular and Life Sciences Curtin University Bentley Western Australia 6102 Australia; ^30^ Hawkesbury Institute for the Environment Western Sydney University Richmond New South Wales 2753 Australia; ^31^ Research Institute for the Environment and Livelihoods Charles Darwin University Ellengowan Dr Casuarina Northern Territory 0810 Australia; ^32^ Invasive Species and Environmental Biosecurity, Department of Primary Industries and Regional Development 1 Nash St Perth Western Australia 6000 Australia; ^33^ Harry Butler Institute, Murdoch University 90 South St Murdoch Western Australia 6150 Australia; ^34^ School of Agriculture, Food and Ecosystem Sciences The University of Melbourne 4 Water St Creswick Victoria 3363 Australia; ^35^ School of Life and Environmental Sciences The University of Sydney Sydney New South Wales 2006 Australia; ^36^ Northern Tablelands, Local Land Services 126 Taylor Street Armidale New South Wales 2350 Australia; ^37^ Biodiversity and Conservation Science, Department of Biodiversity, Conservation and Attractions Woodvale Western Australia 6026 Australia; ^38^ Natural Resources and Environment Tasmania, Tasmanian Government Hobart Tasmania Australia; ^39^ Queensland Parks and Wildlife Service, Department of Environment Science and Innovation PO Box 1442 Toowoomba Queensland 4350 Australia; ^40^ NSW National Parks and Wildlife Service, NSW Department of Climate Change, Energy, The Environment and Water 4 Parramatta Square, 12 Darcy Street Parramatta New South Wales 2150 Australia; ^41^ Centre for Sustainable Agricultural Systems University of Southern Queensland 487‐535 West Street Darling Heights Queensland 4350 Australia; ^42^ Global Wetlands Program, Coastal and Marine Research Centre, Australian Rivers Institute Griffith University Nathan Queensland 4111 Australia; ^43^ Flora and Fauna Division, Northern Territory Department of Environment, Parks and Water Security Darwin Northern Territory Australia; ^44^ WWF‐Australia Suite 3.01, Level 3/45 Clarence St Sydney New South Wales 2000 Australia; ^45^ Fenner School of Environment and Society, ANU College of Science The Australian National University Canberra Australian Capital Territory 2601 Australia; ^46^ School of Biological, Earth and Environmental Sciences University of New South Wales Sydney New South Wales 2052 Australia; ^47^ Science and Conservation, Bush Heritage Australia Level 10, 637 Flinders Street Docklands Victoria 3008 Australia; ^48^ Research Centre for Future Landscapes, School of Agriculture, Biomedicine and Environment La Trobe University Kingsbury Drive and Plenty Road Bundoora Victoria 3086 Australia; ^49^ Kangaroo Island Land for Wildlife Association Incorporated PO Box 1039 Kingscote South Australia 5223 Australia; ^50^ Tasmanian Land Conservancy 183 Macquarie Street Hobart Tasmania 7000 Australia; ^51^ Biosecurity Queensland, Department of Agriculture and Fisheries, Ecosciences Precinct GPO Box 267 Brisbane Queensland 4001 Australia; ^52^ College of Engineering, Science and the Environment The University of Newcastle University Drive Callaghan New South Wales 2308 Australia; ^53^ Australian Museum Research Institute Australian Museum, 1 William Street Sydney New South Wales 2010 Australia; ^54^ Central‐South Region, Australian Wildlife Conservancy PMB 146 C/O Newhaven Alice Springs Northern Territory 0872 Australia; ^55^ Centre for Mined Land Rehabilitation, Sustainable Minerals Institute University of Queensland Brisbane Queensland 4072 Australia; ^56^ Centre for Tropical Environmental and Sustainability Science, College of Science and Engineering James Cook University Townsville Queensland 4811 Australia; ^57^ Smithsonian Tropical Research Institute Panama Panama; ^58^ Department of Biology Wilfrid Laurier University 75 University Ave W Waterloo ON N2L 3C5 Canada; ^59^ School of BioSciences The University of Melbourne Parkville Victoria 3010 Australia; ^60^ Department of Environment and Genetics, School of Agriculture, Biomedicine and Environment La Trobe University Kingsbury Drive and Plenty Road Bundoora Victoria 3086 Australia; ^61^ ICON Science, School of Global, Urban and Social Studies RMIT University 124 La Trobe Street Melbourne Victoria 3000 Australia; ^62^ Kangaroo Island Landscape Board, Kangaroo Island Landscape Board 35 Dauncey Street Kingscote South Australia 5223 Australia; ^63^ Taronga Institute of Science and Learning, Taronga Conservation Society Australia Bradleys Head Road Mosman New South Wales Australia; ^64^ Threatened Species Operations, Department of Environment Science and Innovation 203 Tor St Toowoomba Queensland 4350 Australia; ^65^ Invasive Species and Environmental Biosecurity, Department of Primary Industries and Regional Development 75 York Rd Northam Western Australia 6401 Australia; ^66^ Centre for Tropical Environmental and Sustainability Science, College of Science and Engineering James Cook University 1/14‐88 McGregor Road Smithfield Queensland 4870 Australia; ^67^ Department of Climate Change, Energy, the Environment and Water, Department of Climate Change, Energy, the Environment and Water GPO Box 3090 Canberra Australian Captial Territory 2601 Australia; ^68^ CIBIO – Research Centre in Biodiversity and Genetic Resources University of Porto Campus de Vairo, Rua Padre Armando Quintas, 4485‐661 Vairo Portugal; ^69^ BIOPOLIS – Program in Genomics, Biodiversity and Land Planning Campus de Vairo, Rua Padre Armando Quintas, 4485‐661 Vairo Portugal; ^70^ Queensland Alliance for Agriculture and Food Innovation University of Queensland Brisbane Queensland 4072 Australia; ^71^ School of Agriculture and Food Sustainability University of Queensland Brisbane Queensland 4072 Australia; ^72^ North‐East Region, Australian Wildlife Conservancy 21 Balfe St Cairns Queensland 4870 Australia; ^73^ Flora and Fauna Division, Northern Territory Department of Environment, Parks and Water Security Arid Zone Research Institute South Stuart Highway Alice Springs Northern Territory 0870 Australia; ^74^ Nature Foundation Level 2, Payinthi, 128 Prospect Road, Prospect South Australia 5082 Australia; ^75^ Savanna Field Station Belize District La Democracia Belize; ^76^ Vertebrate Pest Research Unit, NSW Department of Primary Industries and Regional Development 30 Park Avenue Coffs Harbour New South Wales 2450 Australia; ^77^ Natural Environments Program, Gippsland, Victorian Government Department of Environment, Land, Water and Planning 71‐173 Nicholson Street, Orbost Victoria 3888 Australia; ^78^ School of Natural Sciences Macquarie University, Wallumattagal Campus Macquarie Park New South Wales Australia; ^79^ Future Regions Research Centre Federation University Mount Helen Victoria 3350 Australia; ^80^ Australian Landscape Trust PO Box 955 Renmark South Australia 5341 Australia; ^81^ Minesite Biodiversity Monitoring with eDNA (MBioMe) Research Group, TrEnD Lab, School of Life and Molecular Sciences Curtin University Bentley Western Australia 6102 Australia; ^82^ Atlas of Living Australia, CSIRO National Collections and Marine Infrastructure Melbourne Victoria Australia; ^83^ College of Science and Engineering James Cook University Townsville Queensland 4811 Australia; ^84^ Gadfly Ecological Services 34 Ashford Street Shorncliffe Queensland 4017 Australia; ^85^ Terrestrial Ecosystem Research Network The University of Adelaide GPO Box 498 Adelaide South Australia 5005 Australia; ^86^ School of Science Western Sydney University Richmond New South Wales 2753 Australia; ^87^ School of Natural Science, Faculty of Science and Engineering Macquarie University Wallumattagal Campus Macquarie Park New South Wales Australia; ^88^ School of Biological Sciences University of Western Australia 35 Stirling Hwy Crawley Perth Western Australia 6009 Australia; ^89^ Environment, CSIRO Underwood Ave Floreat Western Australia 6010 Australia; ^90^ School of Veterinary Science University of Queensland Gatton Queensland Australia; ^91^ Australian Pet Welfare Foundation 13 Robertson Place, Fig Tree Pocket Queensland 4069 Australia; ^92^ Health and Biosecurity, Commonwealth Science and Industrial Research Organisation Brisbane Queensland Australia; ^93^ Threatened Species Operations, Department of Environment Science and Innovation Level 6, 12 First Avenue Maroochydore Queensland 4558 Australia; ^94^ Queensland Parks and Wildlife Service, Department of Environment Science and Innovation PO Box 44, Innisfail Queensland 4860 Australia; ^95^ Department of Energy, Environment and Climate Action, Arthur Rylah Institute for Environmental Research 123 Brown Street Heidelberg Victoria 3084 Australia; ^96^ Wildlife and Natural Heritage, Royal Commision of Alula Road 375, 7487 Alula Saudi Arabia; ^97^ School of Science, Engineering and Technology University of the Sunshine Coast Maroochydore DC Queensland 4558 Australia; ^98^ National Science, Australian Wildlife Conservancy PO Box 8070, East Subiaco Western Australia 6008 Australia; ^99^ Vertebrate Pest Research Unit, NSW Department of Primary Industries and Regional Development Armidale New South Wales 2351 Australia; ^100^ Ecology Team, Stantec Australia Perth Western Australia 6000 Australia; ^101^ School of Environmental and Conservation Science Murdoch University Murdoch Western Australia 6150 Australia; ^102^ Environment, Commonwealth Science and Industrial Research Organisation Winnellie Northern Territory Australia; ^103^ Conservation Department Phillip Island Nature Parks, PO Box 97 Cowes Victoria 3922 Australia; ^104^ School of Biology and Environmental Science Queensland University of Technology Gardens Point Campus Brisbane Queensland 4000 Australia; ^105^ Terrestrial Ecosystems 10 Houston Place Mt Claremont Western Australia 6010 Australia; ^106^ School of Molecular and Life Sciences Curtin University Bentley Western Australia 6102 Australia; ^107^ Environment & Science Directorate, Research Partnerships & Programs Unit Parks Victoria, Level 1, 65 Church Street Morwell Victoria 3840 Australia; ^108^ Greater Sydney, Local Land Services Level 4, 2‐6 Station Street Penrith New South Wales 2750 Australia; ^109^ Southern Ark, Victorian Government Department of Energy, Environment and Climate Action 171‐173 Nicholson Street Orbost Victoria 3888 Australia; ^110^ Wildlife Conservation and Science Department, Zoos Victoria Healesville Sanctuary, Badger Creek Road Healesville Victoria 3777 Australia; ^111^ Deakin Marine Research and Innovation Centre Geelong Deakin University Geelong VIC Australia

**Keywords:** Australia, terrestrial vertebrates, biodiversity conservation, data sharing, sampling methods, occupancy modelling, big data

## Abstract

Camera traps are widely used in wildlife research and monitoring, so it is imperative to understand their strengths, limitations, and potential for increasing impact. We investigated a decade of use of wildlife cameras (2012–2022) with a case study on Australian terrestrial vertebrates using a multifaceted approach. We (*i*) synthesised information from a literature review; (*ii*) conducted an online questionnaire of 132 professionals; (*iii*) hosted an in‐person workshop of 28 leading experts representing academia, non‐governmental organisations (NGOs), and government; and (*iv*) mapped camera trap usage based on all sources. We predicted that the last decade would have shown: (*i*) exponentially increasing sampling effort, a continuation of camera usage trends up to 2012; (*ii*) analytics to have shifted from naive presence/absence and capture rates towards hierarchical modelling that accounts for imperfect detection, thereby improving the quality of outputs and inferences on occupancy, abundance, and density; and (*iii*) broader research scales in terms of multi‐species, multi‐site and multi‐year studies. However, the results showed that the sampling effort has reached a plateau, with publication rates increasing only modestly. Users reported reaching a saturation point in terms of images that could be processed by humans and time for complex analyses and academic writing. There were strong taxonomic and geographic biases towards medium–large mammals (>500 g) in forests along Australia's southeastern coastlines, reflecting proximity to major cities. Regarding analytical choices, bias‐prone indices still accounted for ~50% of outputs and this was consistent across user groups. Multi‐species, multi‐site and multiple‐year studies were rare, largely driven by hesitancy around collaboration and data sharing. There is no widely used repository for wildlife camera images and the Atlas of Living Australia (ALA) is the dominant repository for sharing tabular occurrence records. However, the ALA is presence‐only and thus is unsuitable for creating detection histories with absences, inhibiting hierarchical modelling. Workshop discussions identified a pressing need for collaboration to enhance the efficiency, quality and scale of research and management outcomes, leading to the proposal of a Wildlife Observatory of Australia (WildObs). To encourage data standards and sharing, WildObs should (*i*) promote a metadata collection app; (*ii*) create a tagged image repository to facilitate artificial intelligence/machine learning (AI/ML) computer vision research in this space; (*iii*) address the image identification bottleneck *via* the use of AI/ML‐powered image‐processing platforms; (*iv*) create data commons for detection histories that are suitable for hierarchical modelling; and (*v*) provide capacity building and tools for hierarchical modelling. Our review highlights that while Australia's investments in monitoring biodiversity with cameras position it to be a global leader in this context, realising that potential requires a paradigm shift towards best practices for collecting, curating, sharing and analysing ‘Big Data’. Our findings and framework have broad applicability outside Australia to enhance camera usage to meet conservation and management objectives ranging from local to global scales. This review articulates a country/continental observatory approach that is also suitable for international collaborative wildlife research networks.

## I. INTRODUCTION

### (1) The need for wildlife monitoring at large spatial and temporal scales

Accurately and affordably sampling and monitoring wildlife populations is crucial for addressing the biodiversity crisis and managing invasive species but challenging for many species (Dinerstein *et al*., 2019; Leclere *et al*., 2020). Large‐scale or long‐term monitoring is only common for the most charismatic (e.g. koalas *Phascolarctos cinereus*) or valuable species (e.g. game species for hunters; Likens & Lindenmayer, 2018), and high temporal‐resolution data are limited to easily accessible areas (Jetz *et al*., 2019). For most species and locations, data are sparse and often limited to distributed networks of contributors such as online biodiversity crowd‐sourcing platforms like the Global Biodiversity Information Facility (GBIF), iNaturalist, Ocean Biodiversity Information System (OBIS), and eBird (Mesaglio & Callaghan, 2021; Sullivan *et al*., 2009; Telenius, 2011). While these platforms provide useful presence‐only observations for range mapping and some species distribution modelling frameworks, repeated sampling with consistent protocols is needed for quantifying population dynamics [e.g. occupancy, abundance, and density (Guisan *et al*., 2013; Jetz *et al*., 2019; Leung *et al*., 2020)]. Numerous professional research networks promote standardised monitoring programmes to meet this challenge (Jansen *et al*., 2014; Enetwild‐consortium *et al*., 2023; Casaer *et al*., 2019), as well as initiatives that blend citizen science and standardised sampling, such as ‘Snapshot USA’ or Birdlife Australia's Birdata programme (Baker, Clarke & McGeoch, 2019; Cove *et al*., 2021). However, there remain very few data suitable for terrestrial vertebrate population monitoring in many regions, including Australia.

### (2) Cameras for sampling wildlife

There are numerous strategies for observing free‐ranging terrestrial animals in natural settings. Traditional human visual observations can be challenging or time‐consuming when animals are rare or cryptic, which is becoming more problematic as wildlife populations continue to dwindle (Field, Tyre & Possingham, 2005; Moore *et al*., 2023; Robinson *et al*., 2018). Physically capturing species using traps can be labour‐intensive and faces limitations in ensuring wildlife welfare (Waudby, Petit & Gill, 2019). Salaries for field staff can make these approaches prohibitively expensive.

A variety of stakeholders and users from across the globe have increasingly turned to non‐invasive surveying techniques using passive sensors such as acoustic monitors, drones, and wildlife cameras. While we focus on wildlife cameras in this review, many of the themes and issues will be similar across disciplines of passive monitoring, including eco‐acoustics and environmental DNA (eDNA). Wildlife cameras are triggered by passive or active infrared heat‐in‐motion sensors (also called camera traps or sensor cameras), hereafter referred to as ‘cameras’ (Kays *et al*., 2020) (see Table 1 for glossary). Cameras feature prominently in field‐based monitoring programs that generate data to understand animal abundance, distribution, diversity, survival, and behaviour. Cameras are used extensively in applied contexts to monitor the effectiveness of conservation interventions and pest species management, track ecosystem responses to threats like climate change, fires or disease, or to inform and reduce the environmental costs of human impacts and economic developments such as mining (Greenberg, Godin & Whittington, 2019; Kays *et al*., 2020; Meek *et al*., 2015).

**Table 1:** Glossary.

**Wildlife camera**	Also called camera traps, game cameras, trail cameras, sensor cameras, or field cameras. A device specifically designed to capture images of wildlife without a human present. Most commercially available cameras use a passive infrared sensor to detect when there are variations in both temperature and movement.
**Detectability**	The probability that a species will be detected, if it is present at a site, using a defined survey method.
**Detection history**	A matrix describing the captures through time per species and per site, with cells populated by either binary data (presence–absence zeros and ones) or counts per detection period.
**Hierarchical modelling**	In the context of wildlife camera analysis, this is a statistical approach used to analyse detection histories to derive animal population parameters while accounting for imperfect detection. The key innovation of hierarchical modelling is simultaneously modelling the process of animal presence – sometimes referred to as ‘state variables’ such as occupancy, abundance, or density – and adjusting this estimate based on estimated detection probability. Hierarchical modelling addresses the limitations and biases associated with the detection of wildlife in the field, often leading to more robust and informative results.
**Site**	Small area (less than *ca*. 1 km^2^) with a single or cluster of camera deployments. For example, in mark–recapture studies, paired cameras at a single point are used to capture markings on both sides of a predator, and paired or clustered setups are used to sample nearby on‐ and off‐trail locations simultaneously.
**Landscape**	Contiguous area (*ca*. 1 km^2^ to *ca*. 1000 km^2^) where multiple camera sampling sites are grouped into a survey.
**Survey**	A synchronous deployment of >10 cameras spaced at >0.1 km intervals and left for >10 days. Many surveys are designed to produce detection histories that are suitable for multiple types of analysis, including species accumulation curves or hierarchical modelling, and include metadata that describes the site characteristics and sampling (e.g. coordinates of each camera, elevation, camera placement on or off a trail or road, baiting).
**Study**	A study could include data from multiple surveys, such as comparing results from surveys of the same site across multiple years or comparing results from surveys in different protected areas.
**Monitoring programmes**	A series of surveys utilising similar methods with the objective of understanding how the population of a target species or community changes through time.

Cameras have logistical advantages over conventional techniques that require physical capture or direct observation. Cameras produce objective, verifiable, archivable data sets. Cameras can be active 24 h a day and readily detect elusive and cryptic animals, especially in disturbed habitats where animals are generally more nocturnal or increase their flight distance, reducing direct sightings (Gaynor *et al*., 2018; Tobler *et al*., 2008). Arguably, one of their greatest strengths is that they can also be deployed for long periods (weeks to months), allowing potentially higher detection rates for particular species, e.g. long‐nosed potaroo (*Potorous tridactylus*), long‐nosed bandicoot (*Perameles nasuta*), and southern brown bandicoot (*Isoodon obesulus*) (Claridge, Paull & Welbourne, 2019). Cameras can be deployed in a targeted manner to monitor an individual species, but still usually gather substantial amounts of ‘by‐catch data’ detecting species that were not the initial target of the survey (Henderson *et al*., 2022), or they can be explicitly deployed to monitor a large proportion of terrestrial vertebrate species within a wildlife community; thus a single survey can be used to answer multiple research or monitoring questions (Kays *et al*., 2020; Kelly & Holub, 2008). As camera data have become more prevalent, the number of different analytical approaches available has also proliferated, enabling robust approaches to assess diversity, occupancy, density, abundance, behaviour, and interactions between species (Sollmann, 2018).

Cameras also have disadvantages when considering their use for large‐scale, long‐term terrestrial wildlife monitoring, especially where it is desirable to identify, take a sample from, or closely examine individuals. Limitations compared to live trapping and direct observations include the inability or difficulty to monitor ectothermic taxa (Corva *et al*., 2022), reliably identify individuals within a species (Dorning & Harris, 2019), assess demographic characteristics such as sex or breeding status (Dheer *et al*., 2022), and collect samples for genomic approaches, health surveillance or disease prevalence monitoring (Driessen *et al*., 2021; Hohnen *et al*., 2019). Specific taxa present unique difficulties; for example, identifying between morphologically similar species, such as small rodents, can be challenging, combined with their smaller body size leading to them being less likely to be detected by cameras (Meek & Vernes, 2016; Potter, Brady & Murphy, 2018; Kays *et al*., 2022). Small mammal detectability can be improved through custom deployment strategies like downward‐facing white flash cameras, although this renders cameras less suitable for surveying large mammals (Meek & Vernes, 2016). Programmes seeking to monitor different‐sized animals could adopt both methodologies in paired deployments, which would result in a doubling of required cameras to maintain the same total spatial coverage. Similarly, cameras placed on roads and trails predominately capture predators (e.g. dingoes, foxes, and cats), while cameras in the habitat are more likely to detect native mammals. Monitoring the entire terrestrial mammal community thus requires three cameras, one on roads or trails and two in the habitat, with one of the habitat cameras angled downwards.

Further, in practice, camera images are easier to acquire than to process and analyse, albeit with the caveat that this is predominantly a historical problem with technological advances (Glover‐Kapfer *et al*., 2019; Greenberg *et al*., 2019). A single study may produce millions of images that must be annotated to the level of species or individual – a laborious, expensive process that bottlenecks research and decision‐making (Ahumada *et al*., 2020). While artificial intelligence (AI), machine learning (ML), and collaborative data sets [e.g. eMammal and ClassifyMe (McShea *et al*., 2015; Falzon *et al*., 2019)] have sped up the conversion of images into data sets, human oversight is still necessary, albeit with diminishing intensity, as data sharing of labelled images increases (Vélez *et al*., 2022; Whytock *et al*., 2021). Image‐processing platforms such as Wildlife Insights use detectors like MegaDetector and computer vision image classifiers to identify species, facilitating users' ability to sort, organise, and store images and produce standardised spreadsheets for analysis (Ahumada *et al*., 2020; Beery, Morris & Yang, 2019; Harris *et al*., 2010; Microsoft, 2020). Until recently, most camera data sets have been stored offline, likely due to the cost of some collaborative platforms. Consequently, different projects are hard to integrate into larger data sets, hindering collaboration among camera users and organisations. Despite similar camera deployment strategies, this isolation has prevented powerful cross‐site or longitudinal analyses of population trends. Australia is an ideal case study to facilitate large‐scale, long‐term wildlife monitoring with cameras, given its substantial financial investments into monitoring across diverse stakeholders and high levels of access to education and advanced training in wildlife ecology, e.g. the Northern Environmental Science Program Resilient Landscapes projects (NESP, 2023).

### (3) Wildlife cameras in Australia

Australia faces pressing challenges to conserve its native wildlife, given its highest global rates of contemporary mammal extinctions (Bergstrom *et al*., 2021). Key threats include land clearing, invasive species and climate change, the latter increasing stochastic events like floods, droughts and megafires (Legge *et al*., 2023; Woinarski *et al*., 2019). Australia's vast and often rugged and remote landscapes and extreme climates make cameras a safer and more efficient monitoring solution than traditional methods like line distance sampling transects and live trapping (Moore *et al*., 2023; Wearn & Glover‐Kapfer, 2019). Meek *et al*. (2015) reviewed wildlife camera use in Australia and showed a transition from early adopters undertaking small‐scale exploratory studies to broader use in the mainstream research and conservation community between the mid‐2000s and early 2010s. Meek *et al*. (2015) also reported increasing affordability and reliability of camera technology, including longer battery life and larger image storage, and the introduction of robust analytical methods such as hierarchical occupancy modelling (MacKenzie *et al*., 2002).

Today, many Australian stakeholders use cameras extensively, including academics, industry (e.g. environmental impact assessments, environmental consultancies), and government and non‐government organisations (NGOs). Thousands of cameras are deployed across Australia annually, collecting millions of images that could be used for large‐scale, long‐term wildlife monitoring and to enhance environmental and economic outcomes. However, a lack of coordination and synthesis has resulted in duplication of effort instead of larger and richer data sets. The review of camera use in Australia by Meek *et al*. (2015) revealed an increasing trend in published studies and technical reports (from one in 1991 to 19 in 2013), reflecting global camera trends (McCallum, 2013; Speaker *et al*., 2022). Building on this, we quantify the ‘where, what, how, and who’ of camera use across the Australian continent from 2012 to 2022 and identify gaps and opportunities for collaboration. We evaluate the strengths and weaknesses of Australia's most used wildlife camera methods and distributed sampling networks to enable large‐scale, long‐term monitoring of wildlife populations. This forward‐looking synthesis aims to leverage investments in cameras for large‐scale, long‐term wildlife monitoring, including whether a distributed network of data providers would be advantageous.

### (4) Hypotheses about camera trends

We quantify trends in camera deployment strategies, analysis, and reporting/publication across stakeholders at various institutions that use cameras to monitor wildlife populations. Our hypotheses, predictions, results, and methods used are summarised in Table 2. We used four approaches to investigate these questions in Australia: a literature review, an online questionnaire, an in‐person workshop, and a compilation and visualisation of camera use in Australia.

**Table 2:** General hypotheses, specific predictions, data‐collection methods, and results on camera use for large‐scale and long‐term wildlife monitoring. Hypotheses and predictions were drawn or inspired by results from prior work in Australia, other countries or global patterns (e.g. Ahumada *et al.*, 2020; Cove *et al.*, 2021; Greenberg *et al.*, 2019; Kays *et al.*, 2020; Sun *et al.*, 2021; Woinarski *et al.*, 2018). AI/ML, artificial intelligence/machine learning; NGO, non‐governmental organisation.

Hypothesis number	General hypothesis	Specific prediction tested	Method	Result
H1	Cameras perceived as the best wildlife sampling approach for cost‐effectiveness and reliability for monitoring terrestrial vertebrates.	Participants' ranking of cameras will be higher than for traditional methods such as live trapping and direct observations.	Questionnaire	Cameras ranked first in cost‐effectiveness (73% first choice) and data quality (88% first choice).
H2a	Exponentially increased camera sampling effort as a result of better technology (longer battery) and lower costs per unit.	Camera sampling will increase through time (the number of studies and cameras and duration deployed per study).	Literature review	Number of surveys – no change. Cameras deployed – no change. Duration cameras deployed – no change.
H2b	Shorter time delay from sampling to publication date due to efficient image processing platforms (AI/ML computer vision in Wildlife Insights) and improved analytical tools (R packages), also facilitating higher publication rates.	Reduced time lag between sampling end date and publication date, more publications per year and publications per sampling effort.	Literature review	Number of publications – linear increase. Time lag – no change.
H3	Different stakeholder/institutional types will sample differently and disseminate findings through different channels.	Government sampling focused on single species to meet regulatory targets (e.g. threatened or invasive species) and disseminate results in internal reports; NGOs focused on managing properties and/or projects with dissemination in internal reports; universities focused on peer‐reviewed publications.	Questionnaire	Government – no significant trends. NGOs – significantly more likely to generate internal management reports based on single species or single sites. University – more likely to generate multi‐species/site peer‐reviewed papers.
H4	Stakeholder/institutional types will dictate the analysis methods used to monitor wildlife, with barriers to using advanced statistical tools that require more quantitative training limiting their use in government and NGOs.	Government will favour more intuitive and easier‐to‐interpret measures such as presence–absence and relative abundance indices; NGOs will favour diversity and relative abundance metrics; university will favour more robust measures such as occupancy and density derived from computationally complex hierarchical models.	Questionnaire	Government – no clear patterns. NGOs – more likely to use presence–absence. University – no clear patterns.
H5	Limited but increasing use of robust detection‐corrected analyses due to a delay in uptake and requiring advanced quantitative training.	An increase in peer‐reviewed studies measuring detection‐corrected occupancy, abundance or density and variation among participants based on affiliation in government, NGO or university.	Questionnaire and literature review	Among affiliations of peer‐reviewed studies, 37% of university, 17% of NGO‐, and 54% of government‐associated authors used detection‐corrected analyses. In the self‐reported questionnaire, 54% of university, 31% of NGO, and 61% of government participants used detection‐corrected analyses.
H6	Improved camera technology and penetration in conservation ecology will pave the way for an increasing diversity of taxa sampled with cameras.	Higher diversity of taxa studied through time, moving from predominantly large, charismatic, and ecologically significant mammals towards smaller and non‐mammalian vertebrates, especially because Australia is a hotspot for small mammal and reptile biodiversity.	Questionnaire and literature review	Limited expansion of cameras to alternative taxa other than mammals, with the traditional medium–large mammals (>500 g) being the most prevalent, but small mammals (<500 g) now receiving more attention.
H7	Biased spatial distribution of surveys towards human population centres and infrastructure and where key charismatic target or invasive species occur.	Camera surveys will occur near urban centres, universities and forested habitats that are biodiversity hotspots.	Questionnaire and literature review	Habitat – most sampling in forests. Distribution of surveys – weak negative correlation with distance to the nearest city and no correlation with human density.

## II. METHODS

### (1) Literature review of wildlife studies using cameras

We conducted a systematic literature review to extract published or publicly available information. We located peer‐reviewed papers, book chapters and grey literature using the software *Publish or Perish*, which searches and indexes publications from *Google Scholar* into a spreadsheet (Harzing, 2007). We first used the search terms ‘camera trap’, ‘research’, and ‘Australia’ in November 2022. A second search in August 2023 used slightly different search terms: ‘camera trap’ OR ‘camera‐trap’ OR ‘remote camera’ OR ‘camera’ AND ‘wildlife’ OR ‘animal’ OR ‘mammal’ OR ‘vertebrate’ OR ‘terrestrial vertebrate’ AND ‘Australia’. The software only allows a maximum of 1000 results per search, resulting in 2000 potential matches to review. We reviewed the search results to identify peer‐reviewed studies on vertebrates in terrestrial habitats. We excluded studies using drone and arboreal cameras, including nest box monitoring, and studies focused on evaluating camera performance, rather than wildlife, and on captive animals. Our focus was camera deployments in larger‐scale monitoring of vertebrate populations, so we excluded smaller‐scale studies, such as pilot studies testing novel camera designs. To do this, we only included studies using ≥10 cameras spaced ≥100 m apart and deployed for ≥10 days in any study area to qualify for further analysis and inclusion. The process for how these metrics were established is reported as online Supporting Information in Table S1. Studies were also excluded if they did not report these fundamental attributes, except for spacing, which was assessed visually if not reported based on maps in the publication. We only retained literature items published between 2012 and 2022. All data management and analyses were conducted in R (R Core Team, 2022).

We note that this review likely misses camera users who rarely publish in peer‐reviewed journals such as private consultancy firms conducting environmental impact assessments. The findings of these surveys are kept confidential, at least until the developer submits their assessment. By encouraging collaboration with private industry and environmental consultants, an Observatory (see Section IV.5) could leverage these rich private data sources and fill in monitoring gaps in Australia while providing advanced analytics to the private sector.

### (2) Questionnaire of camera users

We solicited stakeholder experiences about the use of cameras from 132 camera users working in Australia using an online questionnaire distributed *via* email (UQ ethics permit 2022/HE001653; questionnaire available at: https://www.dropbox.com/scl/fi/94f4l4ev2k1z9vs6y9u5i/WildObs‐Camera‐trap‐participant‐survey‐Google‐Forms_2023_11_14.pdf?rlkey=yy840dyu58j5n5n9buf7bqj5c&dl=0). The questionnaire had 52 questions and sought to understand the motivation for using cameras compared to traditional monitoring methods, the locations of study sites in Australia, which taxa and habitats were surveyed using cameras, and which analysis methods were used for camera trap surveys. We emailed the questionnaire to authors of wildlife camera literature before the list was limited to peer‐reviewed publications targeting vertebrates in terrestrial habitats to reduce potential bias and to include camera users with diverse motivations and backgrounds. We used snowball sampling *via* referrals, where existing participants recruited future participants based on a prior relationship with them to reach as many camera users as possible. When a participant submitted multiple responses to describe variable methods across surveys – such as changing practices as new technology became available – we only retained a single response reflecting their most recent experience.

We assessed the influence of participants' organisations on their preferred outputs and provided four choices: single site/species peer‐reviewed; single‐site/species internal management report; multi‐site/species peer‐reviewed publication; and multi‐site/species internal report. We repeated the same process for the type(s) of analyses used: presence/absence; relative abundance indices; hierarchical occupancy modelling; or density estimates. If participants identified that they worked for multiple organisations, their responses were assigned to both.

### (3) Expert working group

We hosted an in‐person workshop over three days dedicated solely to assessing and improving the use of cameras for Australian wildlife research, monitoring, and management. The working group participants represented key Australian wildlife research, conservation and management initiatives across academia, NGOs, and government. The 28 attendees included the directors of government environmental departments, such as those focused on conservation and invasive vertebrate management, Chief Executive Officers and regional managers of conservation NGOs and environmental companies, and academics (Table S2). Other stakeholders representing a diversity of organisational backgrounds were included to solicit their experiences with cameras. Our speakers, activities, and discussions covered preliminary results from the literature review and questionnaire described herein and discussions of problems, solutions, and vision to leverage Australia's investment in cameras for large‐scale wildlife monitoring.

### (4) Camera contributions from the Atlas of Living Australia (ALA)

We identified potential camera deployments in the Atlas of Living Australia (ALA) – which provides data to the GBIF – using five steps to produce a conservative map of survey locations based on the following data‐filtering process: (*i*) we retained only records of mammals attributed as “Human records”, as this was the most common record type for camera trap data. (*ii*) We excluded aquatic, volant, arboreal, and semi‐arboreal mammals. We acknowledge this could lead to underestimating the number of camera deployments, but we wanted to focus our search on terrestrial wildlife using camera traps. (*iii*) We retained records that contained the term ‘camera’ in any row. (*iv*) We were interested in surveys – sets of camera deployments that are spatially and temporally clustered – not in every individual camera. To identify spatially clustered records, we laid a grid of hexagons with an area of 20 km^2^ each across Australia. We then retained one record representing a ‘potential camera survey’ for each 20 km^2^ hexagon per 100‐day period. In doing this, we assumed that records gathered from nearby cameras at similar times were part of the same survey (although we recognise this would not have always been the case). We chose a random record to represent the survey in that grid, as we are only interested in the presence of the camera deployment rather than in the species it detected. (5) To remove active cameras operated by humans, as opposed to cameras set for weeks to months to monitor species, we excluded ALA records with effort measured in time units less than days (e.g. minutes) and removed records that explicitly stated they were using another sampling protocol that did not show evidence of using a camera trap.

### (5) Data compilation and cleaning

We summarised the temporal trends from the literature review data set using the *dplyr* package in R (Wickham *et al*., 2023). For any summaries of survey statistics, for example, the number of cameras, we removed duplicate records when the same data set was used for multiple publications. When choosing between duplicates, we chose the record published closest to the survey's end date. We calculated the time lag between the completion of data collection and publication using the latest sampling end date. We determined the median values for deployment duration in days, number of cameras, and sampling effort (deployment duration × number of cameras) for each year in which the sampling ended. For each study, if different areas were surveyed, for the total number of cameras, we summed all the cameras deployed across the different sites and assigned it to the most recent year that sampling ended. For the deployment duration and sampling effort, if there were multiple studies in different areas, we retained the value from the survey that had the most recent year that the sampling ended; if there were multiple studies where sampling ended in the same year, we retained the highest value for the metric in question in that year. For the number of cameras, deployment duration, and sampling effort, we treated the year sampling ended as a factor, and log_10_ transformed each metric for plotting.

### (6) Statistical analyses

To test our literature review hypothesis that the number of camera publications would increase through time (linearly or exponentially), we compared a linear and non‐linear regression using Akaike Information Criterion corrected for small sample sizes (AICc) model selection in the package *AICcmodavg* (Mazerolle, 2020). The top model with the lowest AICc value included a linear relationship. To assess motivations and preferences for using cameras in the questionnaire, we calculated the percentage of participants that identified each method as their first, second or third choice for each criterion and used a Likert scale with three points to rank monitoring methods for data quality and cost‐effectiveness (Bryer & Speerschneider, 2016).

We analysed the influence of questionnaire participants' organisations which they self‐identified as either NGO, government, or university, with binomial generalised linear models (GLMs) with odds ratios in the *lme4* package, following Sockhill *et al*. (2022) and Bates *et al*. (2015). Each organisation type was included as a binary predictor variable (0/1) to allow participants to identify multiple types, and they could also use multiple methods.

### (7) Spatial mapping and visualisation

To visualise the distribution of surveys in Australia spatially, we created maps in *ggplot2* (Wickham, 2016). First, we plotted each survey identified from the literature or the questionnaire as a single point, the size of which was scaled to the number of cameras deployed. Then, we overlaid 2,500 km^2^ hexagons across Australia and counted the number of studies from the literature review and questionnaire and separately from the ALA data per 2,500 km^2^ hexagon. We assessed the potential number of surveys conducted across ecoregions in Australia (as defined by Olson *et al*., 2001). Each survey from the literature and questionnaire was assigned to the relevant biome based on their spatial coordinates. The total number of surveys conducted in each biome was then calculated in *dplyr*. If a survey was reported in the questionnaire, the same survey was identified in the literature review, or the same data set was used for multiple publications, only one record was retained to prevent duplicate counts from influencing our summaries.

To understand if human population density (Australian Bureau of Statistics, 2022) or distance to the nearest city influenced the number of surveys, we correlated the number of surveys from both the literature and provided by questionnaire respondents per 2,500 km^2^ hexagons for each variable. To calculate the distance to the nearest city, we used the database of world cities from the *maps* package in R (Becker & Wilks, 2022). We measured the distance from each hexagon to its nearest city using the *sf* package (Pebesma, 2018). We excluded any hexagons with zero surveys for the correlation analyses.

## III. RESULTS

### (1) Slowing growth of wildlife cameras (literature review)

**Fig. 1:**
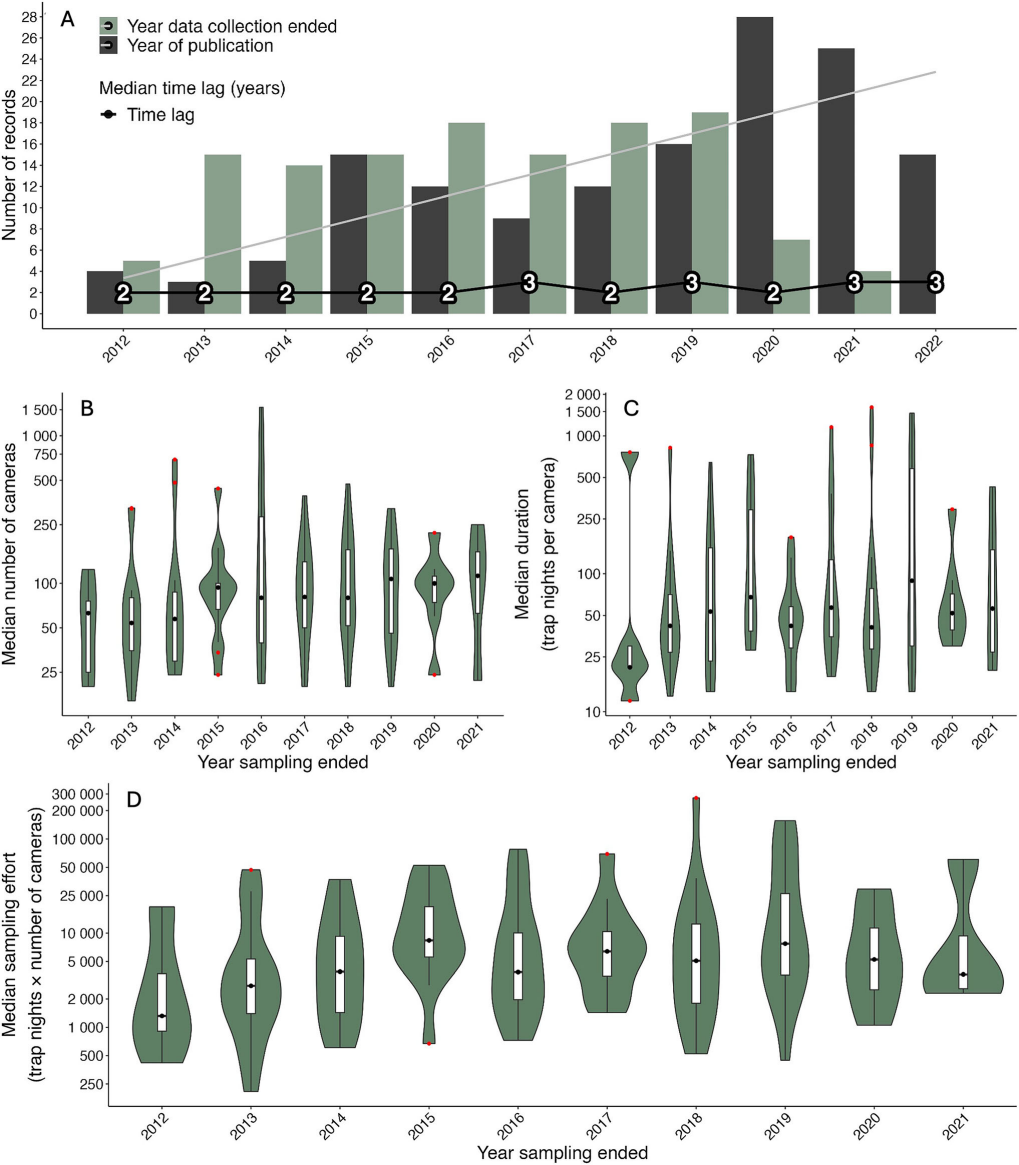
Temporal trends in the use of wildlife cameras in Australia from peer‐reviewed literature. (A) Year of publication (dark grey bars) and the last year of sampling (light green bars). The black line and numbers indicate the median time lag between the year sampling ended and the year the item was published. (B–D) Median effort, which remained highly variable but may have started to plateau at 75–100 cameras deployed per survey that were left for 50–65 days, thus totalling ~5000 total trap nights. All values on the *y*‐axis in (B–D) are log_10_ transformed; black dots in the box plot indicate the median value for the year sampling ended; red points are outliers. Trap nights are calculated as the number of cameras multiplied by duration.

The growth of peer‐reviewed camera studies was modest and linear (coefficient = 1.9455, *t* = 4.014, *p* = 0.003; Fig. 1A), not meeting our predictions of exponential growth. Even more discouraging, sampling effort plateaued from 2013 to 2022 in terms of cameras deployed and total trap nights (Fig. 1B–D). There was also no change in the time lag between data collection and publication. COVID‐19 lockdowns in 2020 did not induce significant changes in publication lag time (Fig. 1A).

### (2) Scale of wildlife cameras research (literature review)

In the literature review of published studies using cameras, 89 (65%) included sampling from a single year, and 47 (35%) included sampling from multiple years or the deployment duration exceeded 365 days. There were 78 studies carried out in a single site (e.g. a national park) and 58 studies carried out across multiple sites. This suggests that two‐thirds of studies focused on a snapshot in time and space (single year, single site), and reveals opportunities to combine single‐site data sets to enable larger‐scale and longer‐term monitoring without substantially infringing on the original authors' aims.

### (3) Motivations for using cameras (questionnaire)

**Fig. 2:**
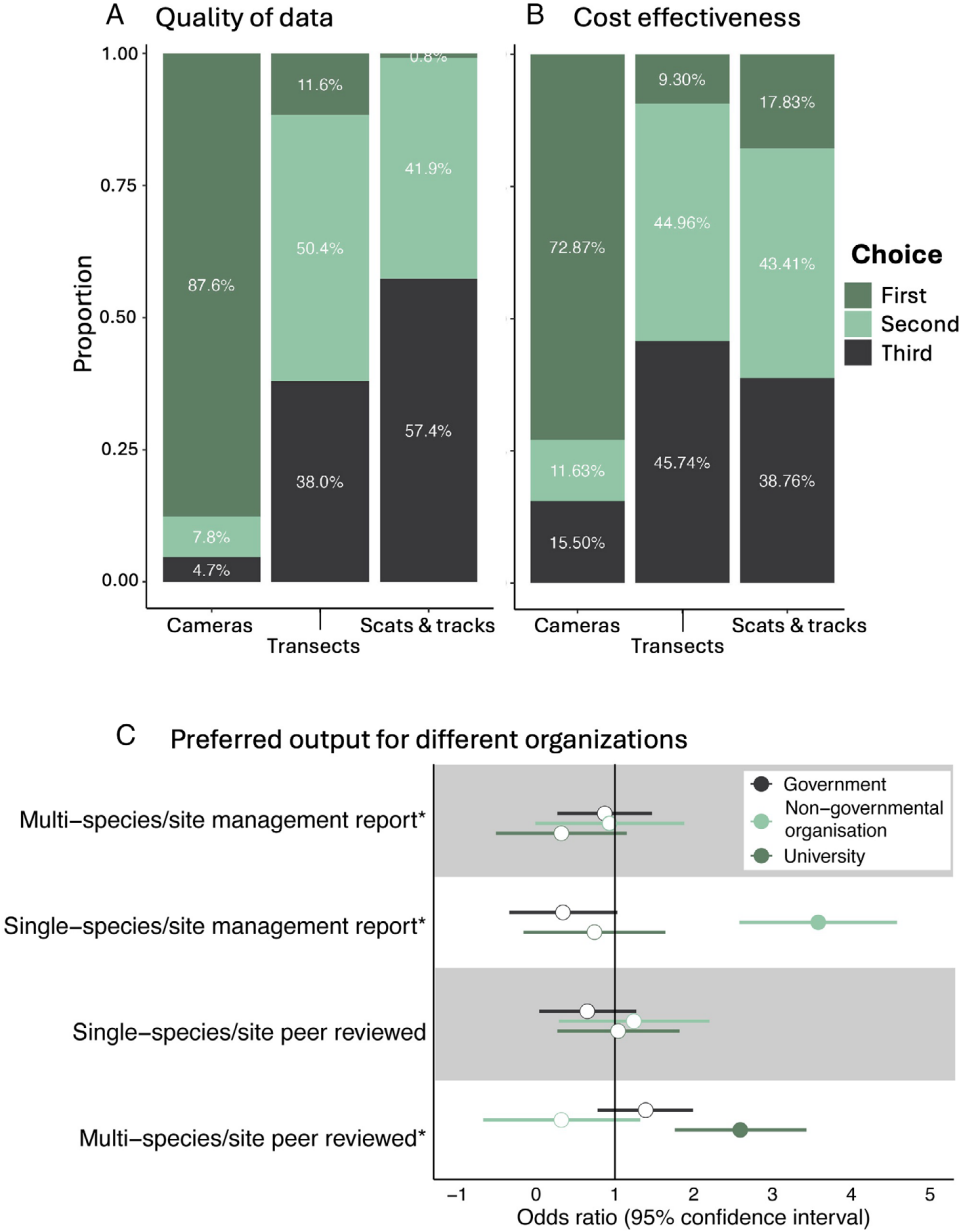
Preferences and motivations for different methods used to sample wildlife. Results show responses from 132 Australian camera users who completed an online questionnaire. Sampling approaches demonstrated a strong preference for cameras regarding (A) data quality and (B) cost‐effectiveness. (C) Results of a binary logistic regression showing odds ratios where values >1 (vertical black line) indicate a positive association (more likely) and <1 a negative association (less likely). An asterisk next to the variable name indicates the overall model was significant using a chi‐squared test. A solid filled point is used where the 95% confidence intervals do not overlap 1.

The questionnaire was completed by 39 government‐, 24 NGO‐ and 60 university‐affiliated camera users, and by nine participants who identified themselves as working across multiple sectors. Cameras yielded the highest data quality regarding reliability and confidence in the data for the least financial investment, with 88% of respondents indicating cameras were their first choice for high‐quality data sets and 73% as the most cost‐effective choice (Fig. 2A, B). ‘Scat, tracks and signs’ was the most prevalent third choice for data quality (57% of responses), and monitoring with transects was seen as the least cost‐effective method (46% of responses; Fig. 2A, B). The motivations for using cameras for university‐affiliated camera users significantly leaned towards multi‐site/species peer‐reviewed publications, compared to NGO‐ or government‐associated camera users (Fig. 2C). NGOs were significantly more likely to generate single species/site management reports (Fig. 2C).

There was little evidence that the use of AI/ML computer vision (CV) to process Australian images is widespread. Only 38/132 respondents indicated they used some form of CV in their image processing. Seven of the 38 respondents who indicated they used CV reported using custom or bespoke software during data processing. Online CV models were the most frequently used CV model, with 30/38 respondents using either a globally trained detection model (MegaDetector) and/or detector plus classifier (Wildlife Insights, which itself uses MegaDetector), or interfaces with MegaDetector (e.g. tools on Ecoassist). Three used Evorta, a for‐profit Australian‐specific online AI model that charges per image.

**Fig. 3:**
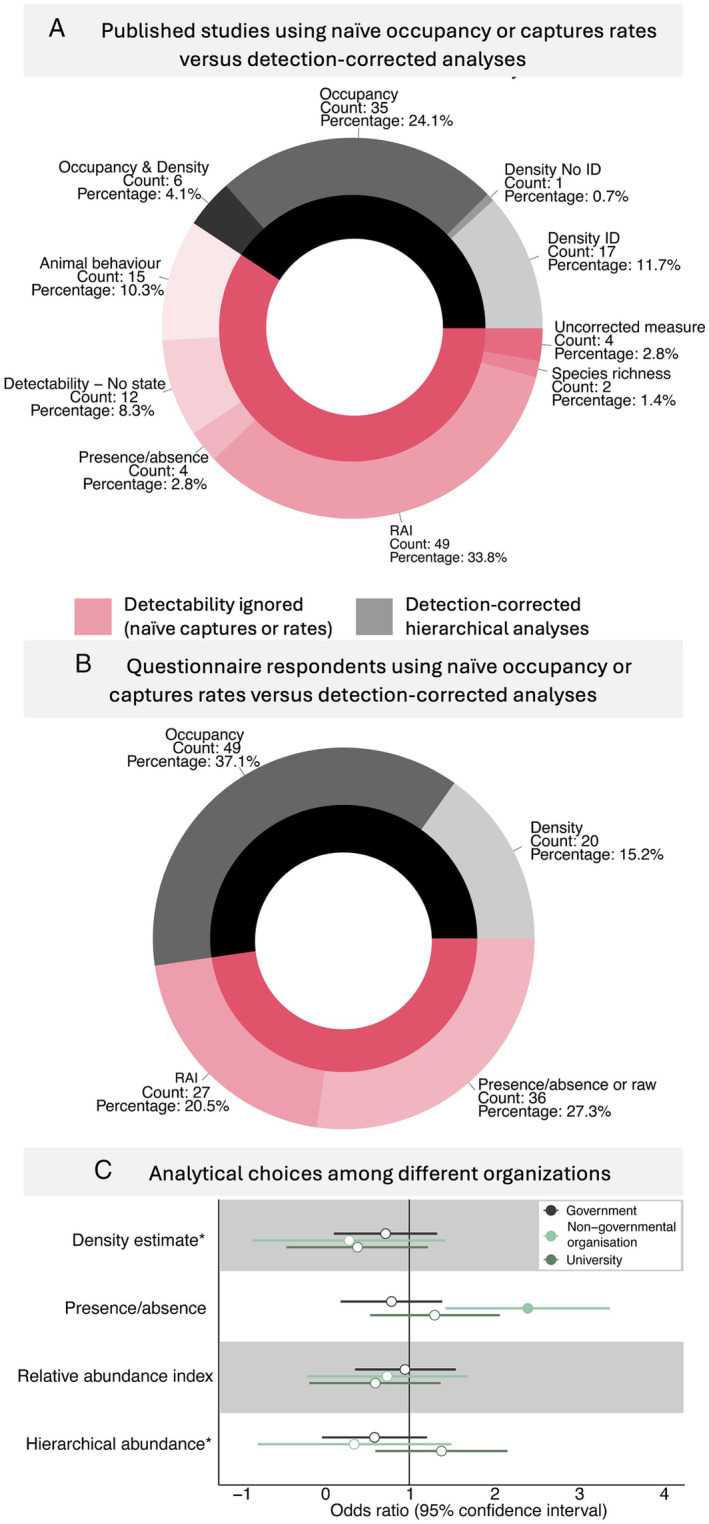
Only half of Australian wildlife camera analyses correct for detection probabilities. (A) Summary of published studies. (B) Responses from 132 Australian camera users who completed an online questionnaire. Methods were grouped by whether they ignored bias associated with the imperfect detection, such as in presence/absence, naïve occupancy (percentage of cameras detecting the species) or capture rates [or their modified forms, such as relative abundance index (RAI) or Allen's index]. Animal behaviour studies were primarily concerned with behaviours like carcass and nest discovery and likely used indices to understand issues such as bait uptake. (C) Results of a binary logistic regression showing odds ratios where values >1 (vertical black line) indicate a positive association (more likely) and <1 a negative association (less likely). An asterisk next to the variable name indicates the overall model was significant using a chi‐squared test. A solid filled point is used where the 95% confidence intervals do not overlap 1.

The questionnaire also revealed that NGO affiliates were more likely to use presence–absence metrics than government or university affiliates (Fig. 3C). There was no significant variation in the other metrics respondents reported they used between the different groups (Fig. 3C). The literature review and the questionnaire found that around half of the respondents and peer‐reviewed papers used analyses that considered detectability (Fig. 3A, B). This pattern varied across organisation types, with 54% of papers with government‐affiliated contact authors accounting for detectability, whereas NGO‐ (17%) and university‐ (37%) affiliated authors had fewer papers considering detectability.

**Fig. 4:**
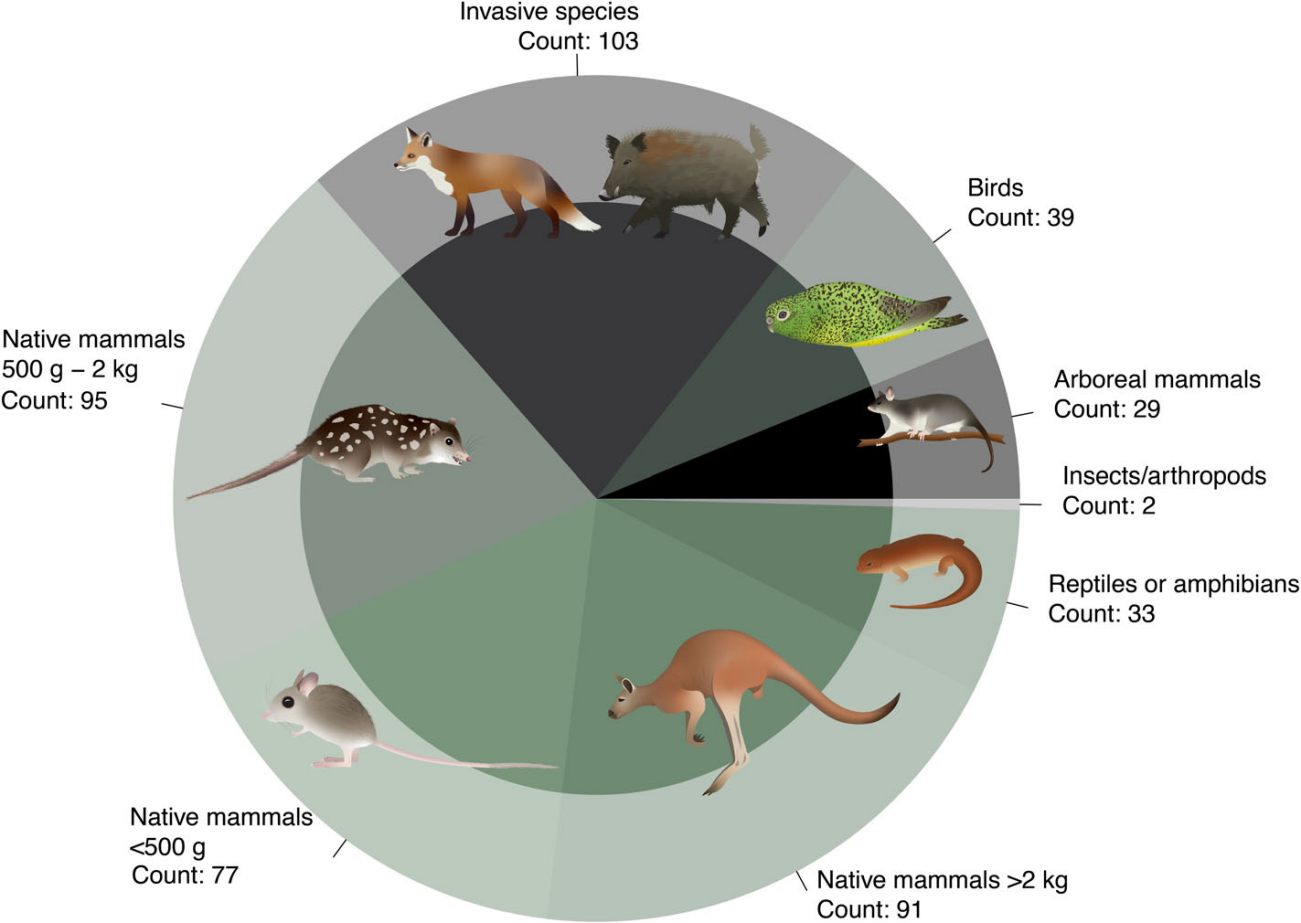
Questionnaire participants indicated which taxa they sampled with cameras, summarized here with larger areas reflecting more users. Animal symbols courtesy of the NESP Resilient Landscapes Hub, nesplandscapes.edu.au.

Respondents to the questionnaire identified that cameras were most often (78% of participants) used to target invasive species and canid predators such as foxes, dingoes, and other wild/domestic/feral dogs. Insects/arthropods received the least use by cameras (1.5%; Fig. 4). Various‐sized native mammals received similar monitoring levels, with native mammals >2 kg monitored by 69% of participants, <2 kg to >500 g by 72%, and < 500 g by 58%. By contrast, reptiles/amphibians, birds, and arboreal mammals garnered less attention, with fewer than 30% of participants reporting using cameras to monitor these taxa.

### (4) Habitats sampled (questionnaire and literature review)

**Fig. 5:**
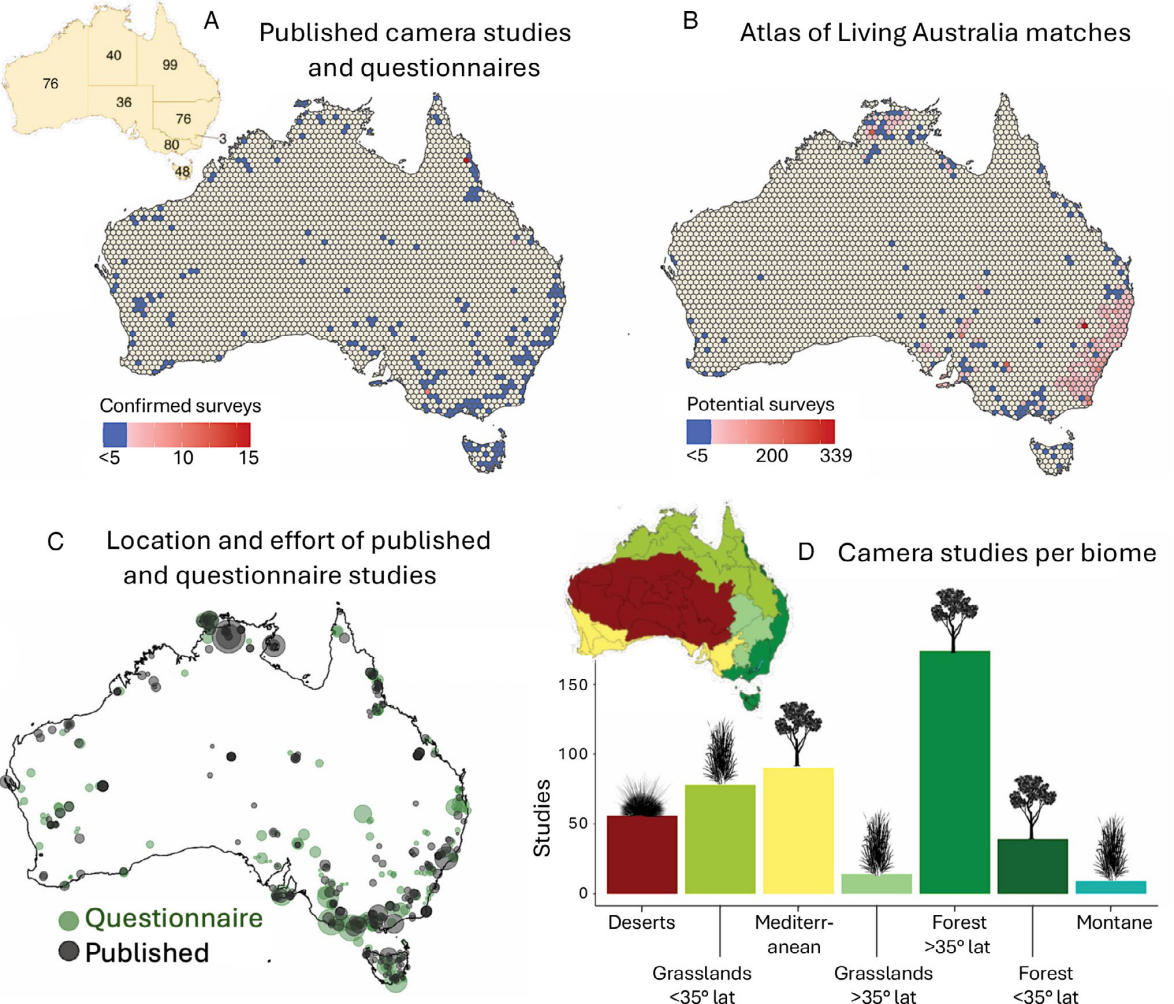
Distribution of camera surveys in Australia. (A, B) Number of camera surveys per 2500 km^2^ hexagonal cell from published literature and questionnaire data (A, with the inset showing values per state), and potential (unconfirmed) sites from the Atlas of Living Australia (B). (C) Published (grey) and questionnaire survey locations (green), with symbol size scaled to the number of cameras reported to be deployed. (D) Published and questionnaire surveys per habitat, ordered from largest area (left) to smallest (right). Above the bars, drawings indicate the biome: trees represent largely forested biomes; grass indicates grassland or savannah; spinifex grass indicates shrub and desert biomes. The inset map shows the spatial distribution of each biome in Australia. Specific biome names from the ecoregion layer, from left to right, are: (*i*) deserts and xeric shrublands; (*ii*) tropical and subtropical grasslands; (*iii*) mediterranean forests; woodlands and scrub; (*iv*) temperate grasslands and savannahs; (*v*) temperate broadleaf and mixed forest; (*vi*) tropical and subtropical moist broadleaf; and (*vii*) montane grasslands and shrublands.

The spatial distribution of camera trap surveys across the literature, questionnaire data, and the ALA data was concentrated along the east coast of Australia, with apparent gaps in the state of Western Australia and large areas of arid Australia (Fig. 5A–C). The number of surveys reported by questionnaire respondents and the literature review was highest in Queensland, with 51 and 48 surveys, respectively, and the lowest was in the smallest territory, the Australian Capital Territory, with three surveys found in the literature, one of which was reported again by a questionnaire respondent. The remaining states and territories had similar numbers of surveys (Table S3). There was a negligible weak negative correlation between the distance to the nearest city and the number of studies conducted in a 2,500 km^2^ hexagon. Despite being the third smallest of Australia's seven biomes (Fig. 5D), the biome with the most camera surveys was temperate broadleaf and mixed forests. Desert and xeric shrublands occupy around 46% of Australia's landmass but were only the fourth most frequently studied biome. There was no clear relationship between the number of surveys and the size of each biome.

### (5) Qualitative findings from the workshop

There was broad agreement during group discussions that building a distributed network of data providers – hereafter an ‘Observatory’ – would efficiently enhance biodiversity monitoring in Australia and has already been successfully implemented in other countries (Enetwild‐consortium, 2024). The key challenges were data sharing amongst different investigators and institutions, with concerns about equitable acknowledgment, intellectual property (IP) rights, and attribution for resulting products and publications. Further, a successful Observatory would require a multidisciplinary team of wildlife ecologists as well as data scientists and experts in the fields of archiving data, technology and digital infrastructure, the latter being a strength of Australia's federally funded Terrestrial Ecosystem Research Network (TERN) that operates other earth observatories. Throughout the discussions, Traditional Owners and their needs were central. Another theme was that Australian camera users often embarked on bespoke deployment strategies, such as baiting or placement in specific habitats (e.g. facing wombat burrows), which strongly dictates detectability. Thus, any Observatory must account for and accommodate diverse sampling strategies, which requires standardised metadata on deployment details. A key concern for an Observatory was the ongoing funding necessary to operate across a longer temporal scale than most grants and funding cycles. For an Observatory to have its desired impact, it must identify funding beyond these avenues and thus be better suited for inclusion in permanent governmental infrastructure, for example, by ensuring standardised indices are mandatory in the State of Environment reports.

### (6) Steps towards large‐scale and long‐term monitoring with wildlife cameras

**Fig. 6:**
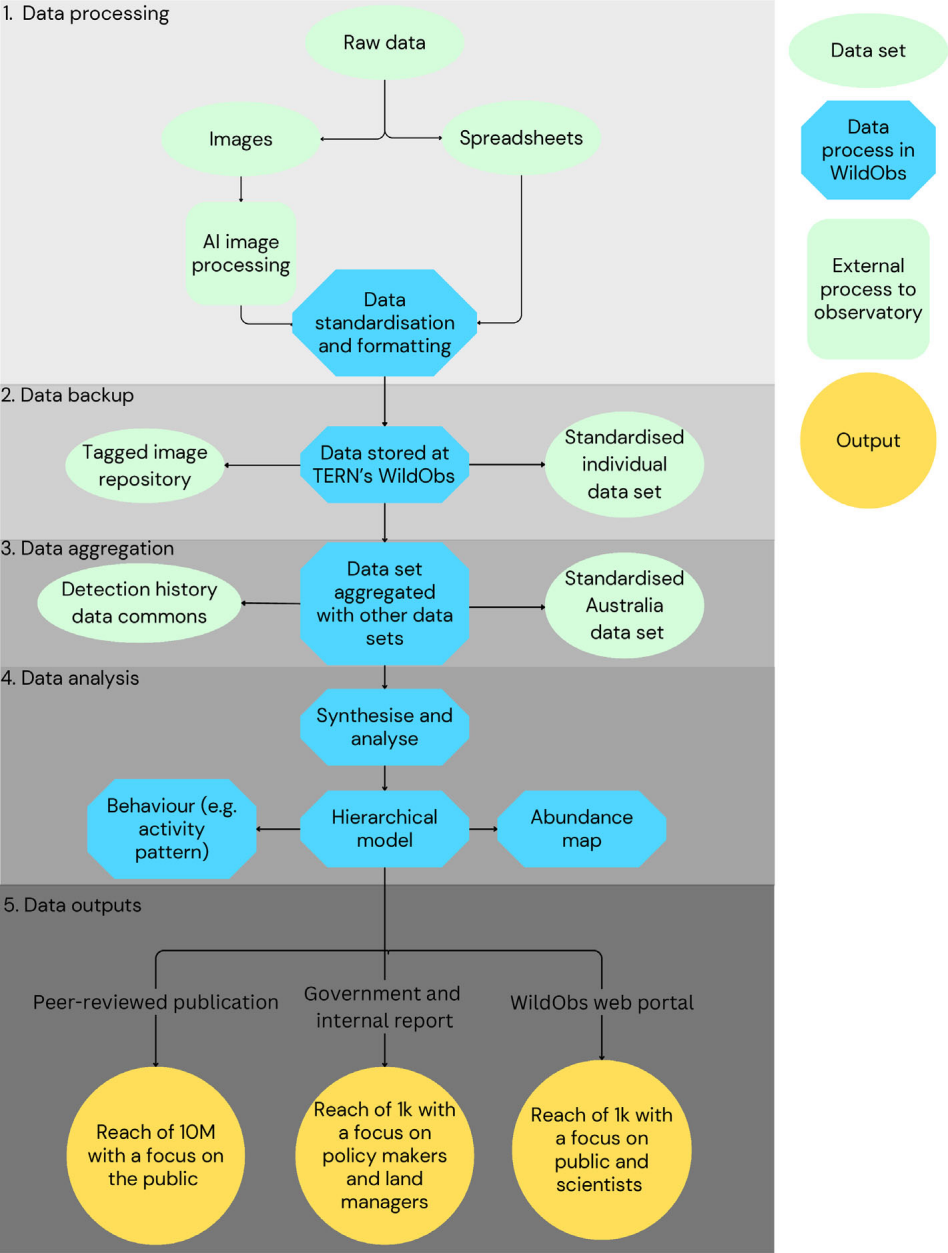
Framework for a distributed network of wildlife camera data providers and its outputs. Data are ingested as raw images or spreadsheets, then standardised, collated, and stored permanently as a data commons, located in existing trusted data providers such as the Atlas of Living Australia (ALA) and the Terrestrial Ecosystem Research Network (TERN). ALA and TERN must add new functionality to store and share detection histories as opposed to presence‐only data. The data are then analysed using hierarchical modelling on high‐performance computing clusters (HPCC) with open‐access code hosted on GitHub. This transparent workflow scales rapidly and is designed for timely outputs for government or NGO reports or peer‐reviewed publications. Data contributors are updated with biannual newsletters and collaboration requests generated between users requesting specific access to data sets. AI, artificial intelligence.

Establishing an effective wildlife Observatory to fill the gaps identified requires the following steps, which are not exhaustive:

(1) Deployment – promote increased standardisation of deployment methods as far as possible and ensure a baseline of appropriate metadata is collected while allowing flexibility to accommodate specific projects. This could be accomplished by providing a free metadata collection app that automatically adds surveys to a national ecology fieldwork registry. Guidance can be provided about how to align ethical and permit requirements for carrying out camera surveys across jurisdictions.
(2) Image storage and processing – promote the use of CV‐powered image management platform(s) to provide image storage, expedite image classification (e.g. species), and export standardised tabular data sets (i.e. spreadsheets for analysis that include the image contents and metadata).
(3) Automated wildlife identifications – facilitate AI/ML CV research in the biodiversity space by providing a tagged image repository. More robust Australia‐specific CV algorithms can be trained using data from a broader range of biomes and species, resulting in increased precision of species identifications and reduced levels of human oversight as the WildObs user base grows.
(4) Data standards – create a national wildlife data commons (or Observatory) with data standards that enable the production of detection histories and adhere to FAIR (Findable, Accessible, Interoperable, and Reusable) data principles (Fig. 6): (*i*) enabling viewing of maps where surveys have been conducted to reduce spatial or taxonomic bias and options to submit data and collaboration requests; (*ii*) allowing concealment of sensitive habitats or species locations in public‐facing maps and overviews, with only approved users able to access specific coordinates following the ARDC protocol (ARDC, 2021); and (*iii*) providing transparent and secure storage for contributed images and spreadsheets and efficient means to contribute and download spreadsheets.
(5) Analytics – provide capacity building (training) and hierarchical modelling tools to monitor population trends while accounting for differences in deployment strategies and detectability.
(6) Reporting – provide intuitive model outputs for the public *via* a web portal and reports for key stakeholders (e.g. Office of the Threatened Species Commissioner).

### (7) Case study: Eyes on Recovery (WWF) and WildCount

**Fig. 7:**
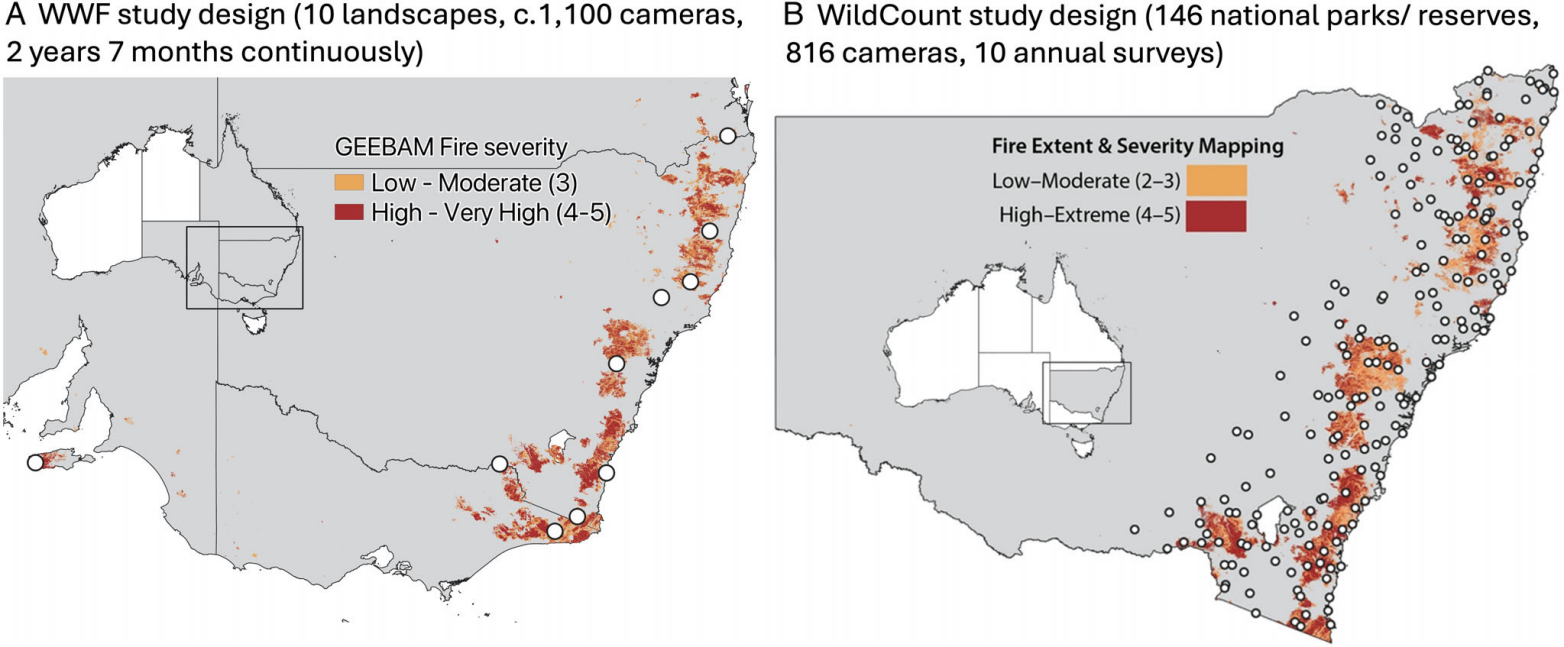
Case studies using cameras for large‐scale long‐term monitoring in Australia. (A) Locations of the 10 major ‘Eyes on Recovery’ study sites denoted by circles with the extent of areas that burnt according to the Google Earth Engine Burnt Area Map (GEEBAM) data set. Low to Moderate burning is represented in orange and High to Very High is in red. (B) Locations of the 204 WildCount monitoring sites denoted by hollow white circles distributed across 146 national parks and reserves in New South Wales; Fire Extent and Severity Mapping (FESM) where burns that were Low to Moderate are represented in orange and High to Extreme are in red (redrawn from Fig. 1 of Lavery *et al*. 2024).

Two monitoring deployments were conducted using very different approaches in New South Wales (NSW). WWF's Eyes on Recovery programme aimed to evaluate impacts and track the status of fauna following the 2019–2020 Australian wildfires, and conducted surveys from November 2020 to June 2023. The programme supported monitoring at nine locations where 17 camera surveys were undertaken in collaboration with 24 on‐ground partner organisations, including government agencies and universities (Fig. 7A). Each survey employed unique deployment strategies based on local‐scale questions, target species, or management objectives. WildCount aimed to monitor wildlife trends across NSW for a decade using a standardised methodology (NSW NPWS, 2020). Across 146 national parks and reserves, each of WildCount's 204 sites were sampled annually from 2012 to 2021, however, each site only used four cameras (Fig. 7B).

Image processing and analytics varied between these two initiatives. WWF managed the collation and processing of images with Wildlife Insights and facilitated the training of Wildlife Insights' global CV model to include Australian species. WildCount did not use publicly available CV‐powered software to process images, although these were unavailable at the project's onset. WWF's on‐the‐ground partners conducted their own site‐level analyses using different approaches but engaged WildObs to analyse the data set as a whole using hierarchical occupancy modelling. WildCount generated standardised five‐ and 10‐year progress reports that employed hierarchical modelling to assess changes in occupancy across years. WildObs provided estimates of occupancy of 56 species through time at all sites. Both programmes carried out workshops following their completion. WWF focused on the logistical challenges of a large deployment and gaining meaningful inference from the distributed network. The WildCount reflections workshop focused on identifying opportunities stemming from its large data set, including using its tagged images to aid developments in ML and AI, improving survey protocols, refining future study sites, and assessing workflows. The WWF results are available in their NGO reporting (https://wwf.org.au/what-we-do/species/eye-on-recovery/) and are in preparation for peer review. Similarly, WildCount provided historical data to contextualise impacts from the 2019–2020 Australian wildfires, available in Lavery *et al*. (2024). The strengths and limitations of these two projects are compared in Table 3.

**Table 3:** Comparison of the strengths and limitations of the approaches to large‐scale and long‐term wildlife monitoring of the WWF ‘Eyes on Recovery’ and the WildCount case studies.

WWF eyes on recovery	
Strengths	Limitations
• Large sampling effort (over 1,100 cameras used), unimpeded by state and protected area boundaries. • Non‐standardised, and more targeted methodology made data relevant and useful for management/research at local scales and for specific species/habitats.	• Reactive, with no existing (or available) pre‐burn data set at almost all survey sites. • Slow to mobilise post‐fire sampling due to delayed access to sites following high‐intensity fires, restrictions on movement and fieldwork during the COVID‐19 pandemic, and time lags for funding, planning, and implementing surveys. • Targeted taxa and camera deployment strategy differed between sites, making analysis of the whole data set more challenging.

NSW WildCount	
Strengths	Limitations
• Long‐term data set (10 years) over a large spatial scale. • Standardised methods for camera deployment maintained across the whole project, both spatially and temporally, allowing the effect of the environment on target species to be more readily quantified. • Proactive monitoring resulted in before‐after–control‐impact opportunities to examine unanticipated, large‐scale disturbances (2019–2020 Australian wildfires). • Standardised species identification methods and assigned levels of identification confidence applied across the programme produced a reliable data set.	• Cameras deployed for a relatively short duration, a minimum 14‐day period. • The intentional design to monitor common fauna species across NSW meant statistical power was lacking to examine occupancy trends among rarer species.

In hindsight, improvements could be made to both approaches to identify locations or species that changed in occupancy (or other metrics) over the last 10 years. For example, WildCount's high coverage but low local effort may be effective at locating areas of concern. This information could then direct additional finer‐scale research – such as the Eyes on Recovery project – to key regions (e.g. a national park) or species of concern. This early warning system was one of the rationales for setting up WildCount. For example, if WildCount detects common species in decline, this may trigger the rollout of finer‐scale studies to investigate the severity and causes of the change.

## IV. DISCUSSION

### (1) Trends in camera use and motivations (H1 and H2)

Most workshop participants and questionnaire respondents considered cameras the most cost‐effective approach to generating high‐quality data sets for monitoring terrestrial vertebrates. This presents a paradox because the growth of Australian camera surveys and publications has slowed over the last decade, especially compared to the decade preceding (Meek *et al*., 2015). Users' rationale for this slowing included logistical issues such as declining investments in environmental space [i.e. reduced staff numbers (Preece & Fitzsimons, 2022; Wintle *et al*., 2019)], increased red tape for camera deployments (e.g. approvals from multiple overlapping jurisdictions), and a general increase in other duties. Importantly, the respondents and participants also suggested this plateau in sampling effort and time lag to publication is partly due to reaching capacity for processing camera images, analysing data, and writing publications. Many people anticipated that camera trap stagnation will not persist if AI/ML‐assisted CV image processing becomes widespread, increasing capacity (Meek *et al*., 2020).

There were several reasons given for why Australia has delayed the widespread adoption of CV to process images and in using collaborative CV‐powered image‐management platforms. First, there were concerns about image ownership and intellectual property security, although this appears unfounded as – to our knowledge – there were no breaches or negative repercussions from using CV tools. Second was doubts about the effectiveness of international open‐access CV models for classifying Australian wildlife, which was deemed generally true as of 2023. Australian camera users also showed a desire to develop and train their own CV models with their own data, rather than using collaborative platforms that leverage the collective skills and data sets from many contributors. This led to Australian CV image‐processing tools being limited to – and replicated within – specific research groups, NGOs, or government departments (e.g. NSW DPI) and the use of local software (Falzon *et al*., 2019; AWC, 2023; Ahumada *et al*., 2020). Things may be changing however, with Wildlife Insights investing in developing the Australian representation in their global CV model and there is now Australian open‐source software for local models (Brook, Buettel & Aandahl, 2023). Addressing this issue should include infrastructure for collaborative CV development such as by hosting a public tagged image repository and providing training, computing expertise, and community for researchers in the CV space. For users focused on ecology *versus* computer science, there is a high demand for free access to a collaborative CV‐powered image‐management platform focused on Australia.

In terms of Australian wildlife camera research analyses and publications, we found around half of the analyses ignored variable species detectability. As many ecologists anticipate the bar for camera publications to increase over time, this may leave one‐off surveys and/or species‐specific work (e.g. university student projects) relegated to more esoteric journals. Established and widely read ecology and conservation journals are shifting their preferences towards multi‐year and multi‐site studies, as well as foci on multi‐species or species interactions, and imposing stricter analytical criteria (e.g. accounting for detectability). Such large and complicated studies take a long time, regardless of the potential increase in CV image‐processing speeds. To enable longer‐term larger‐scale analyses with outputs in management‐relevant timescales may require standardised image and analytical processing systems.

### (2) Variation in the camera analyses among stakeholders/institutions (H3–H5)

Variations in how institutions analyse their camera data sets and distribute their findings have often been discussed anecdotally but rarely quantified. We found relatively small variation in general among academics, NGOs and government, but we also identified opportunities for these user groups to come together for mutually beneficial collaborations. First, all users can increase the scale of inferences and statistical power through data sharing. Second, by sharing complementary resources, such as extensive long‐term government/non‐government data sets (infeasible for academics' limited funding) and academics' comparative expertise in some of the more recent and complex analytics. During the workshop, ample opportunities were identified for NGOs and governments to collaborate with academics. This overcomes two obstacles by leveraging NGOs' and governments' resources for sampling with quantitative expertise in academia, but such collaboration must be encouraged through trust and reciprocally beneficial deliverables. Some key steps towards data sharing include: (*i*) making camera surveys discoverable; (*ii*) improving interoperability by having descriptive and standardised reporting of the methodology; and (*iii*) ensuring camera users are satisfied with how their data are shared and protected, e.g. restricted access to sensitive species, (Bubnicki *et al*., 2023). Using cloud platforms such as Wildlife Insights has improved the discoverability of some data sets, but its use is limited in Australia. A key issue is that biodiversity repositories like GBIF and ALA do not allow for explicit searching for camera surveys. However, there is hope that these issues can be retrospectively addressed because detection histories can still be constructed from historical camera data using standardised data commons, assuming the detections and deployment metadata have been retained.

National Commonwealth grant recipients (e.g. ARC) must submit occurrence data to the ALA, yet enforcement is weak, and submission data standards do not permit the government to use this for many of their desired modelling applications (e.g. occupancy). The issues governments face trying to reuse biodiversity data from publicly funded surveys could be addressed by encouraging grant reporting criteria to have higher standards of data deposition. Expanding data sharing in grant reporting criteria to a broader range of funding agencies (e.g. state‐level, NGO, and private foundation grants) would similarly improve data sharing across academia, government, and NGOs.

### (3) Improving inferences by accounting for detectability (H5)

The metrics used to interpret the findings of camera surveys depended on study objectives, outputs, and target audience preferences. We found only approximately half of published Australian studies and questionnaire participants used analytics that account for detectability in their analyses, often resorting to presence/absence or relative abundance indices (RAI). Both those outputs offer accessible interpretations for diverse audiences but repeatedly have been shown to have systematic biases. Accounting for variable detection is especially problematic when trying to track species population trajectories (e.g. the Threatened Species Index; Bayraktarov *et al*., 2021) or the variables influencing them (Parsons *et al*., 2017). There is a growing scientific consensus around the importance of factoring in detectability for a more robust and nuanced interpretation of camera data sets (Guillera‐Arroita *et al*., 2014), and this issue has previously been raised for Australia (Hayward & Marlow, 2014). This is also relevant in the context of recent natural disasters (e.g. fires) and species undergoing rapid declines driven by multiple factors, some of which are known to affect their detectability. For instance, a study on the New Holland mouse (*Pseudomys novaehollandiae*) demonstrated that decreasing detectability values confounded studies over the past 40 years, rendering previously acceptable search efforts inadequate (Burns *et al*., 2019).

Increasing Australia's use of hierarchical modelling will contribute to delivering more nuanced inferences, but its adoption faces barriers, including access to full detection histories, statistical knowledge, coding skills, and computing power (Ahumada *et al*., 2020; Delisle *et al*., 2021). These challenges may explain why RAIs (which tend to be much more analytically tractable for novice data analysts) remain prevalent despite most questionnaire respondents reporting they understood detectability and wished to account for it. In practical terms, this implies that some Australian wildlife studies and monitoring programmes focusing on population trends may have conflated changes in species detectability and genuine population shifts, posing significant concerns for robust threat assessments and management decisions and interpretations of existing products. This issue is hardly limited to Australia or cameras, including critiques of the Living Planet Index (Buschke *et al*., 2021; Puurtinen, Elo & Kotiaho, 2022). We argue that accounting for detectability is crucial for large‐scale, long‐term monitoring of wildlife populations. The proposed Observatory will remove some significant barriers, like requiring access to advanced statistical knowledge, coding skills, and computing power, by getting users to contribute data and collaborate.

### (4) The distribution of research across taxa, habitats, and the country (H6 and H7)

Camera surveys have historically focused on medium–large terrestrial mammals (>500 g; Delisle *et al*., 2021). When Delisle *et al*. (2021) reviewed how trends in camera use had changed globally from 1995 to 2020, they found that since 2013, publications of studies focusing on birds or herpetofauna have notably increased. This trend has been attributed to subtle changes in camera technology designed to move away from their origins as a tool for big game hunters towards more research‐focused devices and deployment strategies (Ahumada *et al*., 2020; Green *et al*., 2020). We found only equivocal evidence of this trend in Australia, with arboreal mammals, reptiles, and birds being monitored using cameras by less than 30% of questionnaire respondents. The relatively recent development and adoption of vertical‐downward camera‐deployment strategies, which are better at detecting these taxa, could address this in the future. There are also concerns about how reliably smaller animals can be identified at the species level based on infrared imagery alone from horizontally and vertically oriented cameras (Corva *et al*., 2022; Dundas *et al*., 2019; Meek, Vernes & Falzon, 2013; Meek & Cook, 2022).

Addressing biases in camera spatial and taxonomic coverage may be difficult. Globally, 62% of camera surveys occur in forested habitats (McCallum, 2013). This intuitively makes sense because forests' physical structure limits the relative effectiveness of direct observation sampling approaches, and it is easier to hide the cameras relative to more open habitats (Silveira, Jácomo & Diniz‐Filho, 2003). Similarly, deserts and xeric shrubland habitats – Australia's largest ecoregions – were less frequently sampled with cameras than temperate broadleaf and mixed forest ecoregions. Instead, Australia's arid habitats are often sampled using tracks or driven transects. The distribution of surveys also demonstrates a bias towards the country's south‐eastern coast, which has more people and is more accessible (closer to cities, better infrastructure) (Woinarski, Burbidge & Harrison, 2018). Similar large‐scale studies on reptile and bird research in Australia found that proximity to universities, areas with the highest species richness, and sites with high human footprint indices were most likely to be monitored (Piccolo *et al*., 2020; Weston *et al*., 2020). Future studies may struggle to address this spatial bias without also increasing costs for remote deployments.

### (5) The Wildlife Observatory of Australia (‘WildObs’)

A key outcome of the workshop was a commitment to collaboration *via* establishing a Wildlife Observatory (WildObs hereafter) and the launch of WildObs (www.tern.org.au/wildobs). Establishing a distributed network of standardised camera deployments is an established approach, such as the Snapshot USA and the Terrestrial Ecosystem Assessment and Monitoring or ‘TEAM’ network (Jansen *et al*., 2014). What separates WildObs from similar endeavours is the aim to bring together data from all potential sources, regardless of their deployment strategy, as well as support for best practices across deployments, image processing, data curation and sharing, analytics, and reporting (see Section III.6). Large networks like Snapshot USA often impose standardised methodologies to make the data interoperable, which is unsuitable for Australia's reliance on bespoke taxa‐specific deployments. Therefore, WildObs is pursuing an approach to hierarchical modelling where deployment variation is explicitly modelled in the detection function by including deployment covariates (e.g. noting if cameras are sited on a feature like a road or a burrow, the presence and type of bait/lure, camera make and model, etc.). With large sample sizes, this approach allows previously disparate data sets to be analysed through an integrated modelling framework that propagates error (Amir, Sovie & Luskin, 2022).

In the months following WildObs establishment, proof of concept was carried out through collaboration with WWF to analyse their Eyes on Recovery data set to determine the impacts of the ‘Black Summer megafire’ from 2019 to 2020 (see case study in Section III.7 and Table 3). This is one of mainland Australia's most extensive camera endeavours regarding sampling points (1171) and area covered (17 surveys across four states). While large‐scale, longitudinal analyses have been undertaken in Australia for camera data sets using the same deployment strategy (see WildCount; Table 3), Eyes on Recovery permitted several different deployment strategies and thus is effectively a distributed research network.

### (6) Strengths and limitations of a distributed network compared to vertical integration

A key aim of WildObs is to empower camera users to use their data sets to achieve their maximum utility across various applications by making them Findable, Accessible, Interoperable and Reusable (FAIR) (ARDC, 2023). Government and NGO reports, student theses, and the associated data sets generated by these projects are often kept in‐house, making it difficult for other potential users to discover when or where sampling occurred or what was detected. Australia currently lacks a centralised repository for camera data in its full, rich format because existing options – such as ALA and government biodiversity databases – are largely limited to presence‐only or presence–absence data instead of detection histories (ALA adheres to Darwin Core standards). We propose enhancing Darwin Core standards by adding a collection method field, such as transect, camera, or incidental sighting, facilitating rapid filtration by the data source. The incorporation of camera‐specific data standards and criteria outlined in Bubnicki *et al*. (2023), like camera make and model, settings, and deployment details is essential, given their significance in data interpretation. The ALA could then add a dedicated field for querying the data collection methodology (Belbin *et al*., 2021). These changes can enhance the FAIR nature of camera data in existing databases but may not overcome the need for a dedicated data commons with complete detection histories, which could still be hosted at ALA or TERN. A major impediment to users contributing data is the significant time and effort required to format metadata according to strict specifications, which can lead individuals to take shortcuts or omit detailed metadata; WildObs would streamline this using code‐based tools and AI to ensure complete submissions. Contributing to a dedicated camera commons (or database) must meet reporting requirements for government funding and could later be ‘flattened’ to presence–absence or presence‐only data sets to share with the larger biodiversity aggregator databases.

Prior examples of passive sensor networks to monitor wildlife communities are limited in Australia. For example, the Australian Acoustic Observatory (www.acousticobservatory.org) is vertically integrated, essentially a single team conducting long‐term multi‐site monitoring using acoustic recorders and then making the data sets publicly available (Roe *et al*., 2021). The strengths of vertical integration include standardised data sets, while the negatives are the cost of sampling is not shared (buying, deploying, and maintaining all the equipment and data), and this likely limits coverage. By contrast, WildObs will be horizontally integrated with each contributor in charge of their sampling. This enables much larger coverage in space and time, including troves of data already collected over the last decade, but hinges on effective collaboration and data sharing. Another obstacle for horizontal integration to achieve cameras' Big Data potential is the curation and analysis of surveys using different deployment strategies (and doing so at speed). Government investment and/or partnering with technology companies such as Google and Microsoft have proved effective for scaling other environmental networks and platforms (Ahumada *et al*., 2020).

### (7) Wildlife observatories globally

WildCAMS (https://wildcams.ca/) in Canada is a distributed network without the requirement for standardised camera deployments. Alternative distributed network strategies such as TEAM, European Observatory of Wildlife, and Snapshot USA, require standardised camera deployments and can work at the scale of an entire country and globally (Jansen *et al*., 2014; Cove *et al*., 2021; Enetwild‐consortium *et al*., 2023). On the one hand, Australia is relatively well connected given its overall size and could represent the biggest trial in a single country of a standardised initiative globally. Thus far, such standardised deployments in Australia have only been partly implemented for specific projects or individual states like Tasmania or New South Wales (McHugh, Goldingay & Letnic, 2022). On the other hand, many Australian species suffer from low detection probabilities using standardised approaches and thus may require species‐specific deployments to attain sufficient detections to model and monitor them reliably (Henderson *et al*., 2022). Therefore, within Australia, the transition to a standardised deployment is unlikely so WildObs will rely on accounting for this in the detection formula of hierarchical analyses.

## V. CONCLUSIONS

(1) There are immense opportunities for national‐to‐global wildlife monitoring conducted using cameras and this is essential for addressing biodiversity extinction and climate change crises. However, collaborations and innovations are still required to realise the potential of cameras for robust large‐scale long‐term monitoring.
(2) Standardised deployments are impractical for monitoring multiple species across Australia because contributors' immediate reason for sampling is typically species and site specific. There are techniques to overcome these limitations through rigorous data‐cleaning pipelines and hierarchical modelling. Metadata describing camera deployments is a crucial requirement for a distributed network.
(3) The distributed network Observatory approach to data collation – combined with hierarchical modelling analytics – is providing powerful new insights into the ecology and population trajectories of hundreds of species.
(4) The Observatory approach (such as WildObs) offers immense added value to stakeholders that are already undertaking camera surveys for their own reasons, allowing unused data on non‐target species to be analysed. It also connects users with similar aims for collaborations, increasing the cost‐effectiveness of sampling investments and reducing duplication.
(5) Data sharing is crucial to the success of the Observatory model. Trust and incentives can be generated by offering capacity and assistance for image processing, storage, and analysis, and supporting joint efforts for future projects. Many funding agencies already mandate data sharing as part of grant reporting but there are opportunities to increase the effectiveness of this approach.
(6) Providing timely outputs to land managers and decision‐makers (e.g. within weeks) is possible by combining computer vision‐powered image processing with efficient code for data curation and analysis.
(7) The transition from cameras being siloed within teams or organisations to collaborative national wildlife observatories will benefit all stakeholders and enable novel large‐scale long‐term monitoring that is urgently needed.

## Supporting information


**Table S1.** Criteria used to include or exclude a study from the literature review and how the values were derived from the publications.
**Table S2.** Participants of the 2022 workshop and their affiliated institutions.
**Table S3.** Reported number of surveys carried out in each state of Australia, where cameras were the main methodology, found during the literature review or reported by participants in the questionnaire.

## Data Availability

The questionnaire used to investigate stakeholder experiences about the use of cameras is available at: https://www.dropbox.com/scl/fi/94f4l4ev2k1z9vs6y9u5i/WildObs‐Camera‐trap‐participant‐survey‐Google‐Forms_2023_11_14.pdf?rlkey=yy840dyu58j5n5n9buf7bqj5c&dl=0.
