# Supplementary material for: Large‐scale and long‐term wildlife research and monitoring using camera traps: a continental synthesis
Source: Biol Rev Camb Philos Soc. 2025 Jan 17;100(2):530–55. doi: 10.1111/brv.13152 (PMC11885691; doi:10.1111/brv.13152)
Supplement: Supplementary file 1 — Table S1. Criteria used to include or exclude a study from the literature review and how the values were derived from the publications. Table S2. Participants of the 2022 workshop and their affiliated institutions. Table S3. Reported number of surveys carried out in each state of Australia, where cameras were the main methodology, found during the literature review or reported by participants in the questionnaire. [file BRV-100-530-s001.docx]

**Table S1.** Criteria used to include or exclude a study from the literature review and how the values were derived from the publications.

| **Metrics** | **Justification** | **Source** |
| --- | --- | --- |
| Year of publication or production | Year the document was produced or published | Literature review |
| Year sampling ended | The year in which sampling for the literature article finished. This was used to understand the time lag between collection ending and completion. If a study reported multiple end dates, the latest one was used. | Literature review |
| Number of days surveyed | The number of days cameras were deployed, not the total sampling effort. Data were extracted in the hierarchy: (1) Reported average number of days for which cameras were deployed.  (2) Reported maximum number of days if a range is presented.  (3) If a range of dates is presented e.g. 1–20 March, the sum of the days between these dates was used. (4) If the study only presents months, the beginning of the earliest month to the end of the latest month was used (e.g. for March–June 2018, the number of days from 1 March to 30 June was used).  If studies span multiple years then:  (1) If sites are revisited and the same effort is made each year, effort for only one year is summed [e.g McGregor *et al*. (2015) reported a maximum of 6 weeks per site over 3 years; this was recorded as 6 × 7 (= 42) days per year].  (2) For different efforts per year, the year with the largest effort was used.  (3) If the cameras are run continuously the total effort for one camera was used, e.g. 3 years = 365 × 3. | Literature review |
| Number of cameras deployed | The total number of cameras deployed during a survey (e.g. two cameras at 18 sites would total 36 cameras). If there is a range reported and the number is unclear, the minimum number was used.  In the questionnaire, if the participants did not report a number we assumed one camera per site as a conservative estimate. | Literature review |
| Spacing between cameras | The average distance reported between camera traps in kilometres or the minimum distance between camera trap sites. For example, when the minimum distance between sites was reported as 150 m, we recorded it as 0.15. If only grid dimensions were reported these were used to calculate an inter-trap distance assuming equal spacing. | Literature review |

**Table S2.** Participants of the 2022 workshop and their affiliated institutions.

| **Participant** | **Affiliated institution** |
| --- | --- |
| Mr Zachary Amir | University of Queensland |
| Mr Jeff Bell | New South Wales Government |
| Dr Joe Benshemesh | National Malleefowl Recovery Group |
| Mr Colin Broughton | Bush Heritage Australia |
| Professor Diana Fisher | University of Queensland |
| Professor Hedley Grantham | Bush Heritage Australia |
| Dr Aaron Greenville | The University of Sydney |
| Dr Lana Harriot | Department of Agriculture and Fisheries |
| Dr Geoff Heard | University of Queensland |
| Dr Ben Hirsch | James Cook University |
| Dr Malcolm Kennedy | Department of Environment and Science |
| Dr Ashley Leedman | Department of Climate Change, Energy, the Environment and Water |
| Mr Adam McSorley | NSW Department of Primary Industries |
| Dr Paul Meek | NSW Department of Primary Industries |
| Ms Peggy Newman | CSIRO |
| Dr Tom Newsome | The University of Sydney |
| Ms Sally O'Neill | The University of Adelaide |
| Professor Hugh Possingham | University of Queensland |
| Dr Matt Rees | CSIRO |
| Dr Juanita Renwick | Department of Environment and Science |
| Mr Chris Roach | Queensland Parks and Wildlife Service |
| Dr Tracy Rout | WWF-Australia |
| Dr Deane Smith | NSW Department of Primary Industries |
| Dr Emma Spencer | WWF-Australia |
| Dr Rebecca Spindler | Bush Heritage Australia |
| Professor Glenda Wardle | The University of Sydney |
| Dr Alex Watson | Australian Wildlife Conservancy |
| Professor Steve Williams | James Cook University |

**Table S3.** Reported number of surveys carried out in each state of Australia, where cameras were the main methodology, found during the literature review or reported by participants in the questionnaire.

| **State** | **Source** | **Number of surveys** | **Area of state (km^–2^)** | **Records**  **(km^–2^ × 10^3^)** |
| --- | --- | --- | --- | --- |
| Australian Capital Territory | Literature | 3 | 2,358 | 1.27 |
| New South Wales | Questionnaire | 34 | 801,150 | 0.04 |
| New South Wales | Literature | 42 | 801,150 | 0.05 |
| Northern Territory | Questionnaire | 9 | 1,347,791 | 0.01 |
| Northern Territory | Literature | 31 | 1,347,791 | 0.02 |
| Queensland | Questionnaire | 51 | 1,723,030 | 0.03 |
| Queensland | Literature | 48 | 1,723,030 | 0.03 |
| South Australia | Questionnaire | 20 | 984,321 | 0.02 |
| South Australia | Literature | 16 | 984,321 | 0.02 |
| Tasmania | Questionnaire | 10 | 68,401 | 0.15 |
| Tasmania | Literature | 38 | 68,401 | 0.56 |
| Victoria | Questionnaire | 42 | 227,444 | 0.18 |
| Victoria | Literature | 38 | 227,444 | 0.17 |
| Western Australia | Questionnaire | 37 | 2,527,013 | 0.01 |
| Western Australia | Literature | 39 | 2,527,013 | 0.02 |
